# Effects of HLA single chain trimer design on peptide presentation and stability

**DOI:** 10.3389/fimmu.2023.1170462

**Published:** 2023-05-03

**Authors:** Kathryn A. K. Finton, Peter B. Rupert, Della J. Friend, Ana Dinca, Erica S. Lovelace, Matthew Buerger, Domnita V. Rusnac, Ulysses Foote-McNabb, William Chour, James R. Heath, Jean S. Campbell, Robert H. Pierce, Roland K. Strong

**Affiliations:** ^1^ Division of Basic Science, Fred Hutchinson Cancer Research Center (FHCC), Seattle, WA, United States; ^2^ Clinical Research Division, Fred Hutchinson Cancer Center, Seattle, WA, United States; ^3^ Institute for Systems Biology, Seattle, WA, United States

**Keywords:** human class I leukocyte antigens, HLA single-chain trimers, peptide presentation, pan-anti-class I antibodies, X-ray crystallography

## Abstract

MHC class I “*single-chain trimer*” molecules, coupling MHC heavy chain, β_2_-microglobulin, and a specific peptide into a single polypeptide chain, are widely used in research. To more fully understand caveats associated with this design that may affect its use for basic and translational studies, we evaluated a set of engineered single-chain trimers with combinations of stabilizing mutations across eight different classical and non-classical human class I alleles with 44 different peptides, including a novel human/murine chimeric design. While, overall, single-chain trimers accurately recapitulate native molecules, care was needed in selecting designs for studying peptides longer or shorter than 9-mers, as single-chain trimer design could affect peptide conformation. In the process, we observed that *predictions* of peptide binding were often discordant with *experiment* and that yields and stabilities varied widely with construct design. We also developed novel reagents to improve the crystallizability of these proteins and confirmed novel modes of peptide presentation.

## 1 Introduction

**Figure 1:**
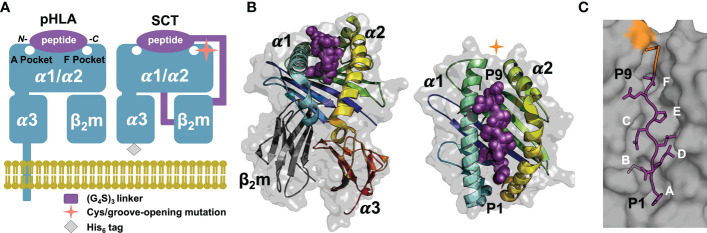
SCT design, structure, and peptide binding. **(A)** Native pHLA and SCT design schematics: the HLA heavy chain α1/α2/α3 and β_2_m domains are shown in *blue*. The linkers incorporated into the SCT design, shown in *purple*, link the C-terminus of the peptide to the N-terminus of β_2_m and the C-terminus of β_2_m to the N-terminus of the HLA heavy chain. The peptide binding pockets, A and F, for the N- and C-terminal peptide residues, respectively are highlighted. While native HLA contains a transmembrane domain, the SCT design is truncated at the C-terminus of the ectodomain and tagged with a 6× histidine purification tag. **(B)** Two views of HLA SCT^H74L/Y84C^ crystal structure (PDB accession code 7SR0) shown as a *gray* transparent surface with an underlying carton ribbon representation, with secondary structure elements indicated. Ribbons are colored as a *blue-to-red* spectrum (*blue*: N-terminus, *red*: C-terminus) for the heavy chain and *dark grey* for β_2_m. The bound peptide is shown as *purple* spheres. **(C)** The SCT peptide binding cleft is shown in a molecular surface representation highlighting the A-F pockets. The YML 9-mer peptide from the SCT^H74L/Y84C^ crystal structure is shown in a *purple* stick representation with p2 (Met) and p9 (Thr) bound in the B- and F-pockets of the HLA binding cleft respectively. Ordered linker residues are colored *orange* in the stick representation and the location of the Y84 cysteine mutation is shown in *orange* on the surface.

The mammalian immune system surveils cellular proteomes to detect intracellular infection or transformation events through recognition of peptide fragments of endogenous proteins bound to cell-surface major histocompatibility (MHC) class I proteins (1). Human MHC class I proteins, or human leukocyte antigens (HLA-I), bind peptides, mostly eight to 14 residues long (2), for presentation on the cell surface for recognition by cytotoxic immune cells (3, 4). HLA-I proteins are composed of a polymorphic, integral-membrane heavy- or α-chain (α1, α2 and α3 ectodomains) and an invariant light chain, β_2_-microglobulin (β_2_m; **Figures 1A, B**) (4, 5). The α1/α2 domains comprise the peptide- and receptor-binding “*platform*” domain featuring a peptide-binding groove. This groove incorporates pockets, labeled A through F, for the N- and C-termini of the peptide and the side chains of “*anchor*” residues, thus determining the peptide specificity of an HLA-I allele (**Figure 1C**).

Recombinantly expressed HLA-I proteins are useful for a variety of applications, including structure/function studies of peptide presentation/specificity, peptide/HLA-I complex (pHLA-I) recognition by immunoreceptors, and for various diagnostic and therapeutic applications (6). The two most widely used platforms for recombinant expression of soluble pHLA-Is are *in vitro* co-refolding (RF) of mixtures of a synthetic peptide and denatured α-chain and β_2_m expressed as bacterial inclusion bodies (pHLA-I^RF^) (7), or secretion from eukaryotic cells. In order to coordinate α/β chain co-expression and incorporate specific peptides during secretion from eukaryotic cells, a linked “*single-chain trimer*” (SCT) construct (8), coupling all three components into a single polypeptide (**Figure 1A**), has been widely used (6). However, use of the SCT design, which incorporates various mutations, may affect peptide presentation and immunorecognition by potentially allowing peptides to shift binding register in the groove by removal of C-terminal bounding constraints, by altering the stability, and therefore peptide binding, of particular pHLA-I complexes, or by enabling unintended disulfide formation between cysteines present in the peptide and engineered cysteines in the SCT.

To more fully understand caveats associated with SCT engineering that may affect their reliability for basic and translational studies, we evaluated a set of engineered SCTs across eight different classical and non-classical HLA-Is with combinations of Y84A, Y84C/linker C, Y84C/A139C, and H74L mutations, with 44 different peptides spanning octamers (8-mers) to tetradecamers (14-mers) including eight peptide-matched and 13 unique-peptide pHLA-I^RF^s.; **Supplementary Table 1**). Biochemical and structural studies of SCT designs showed that SCTs generally recapitulate HLA-I structural and functional features. However, for both SCTs and pHLA-I^RF^s, *predictions* of peptide binding were often discordant with *experiment* and recombinant yields and solution thermostabilities varied widely with construct design. Native peptide conformation could be altered by the SCT mutations employed, and certain SCT mutations were so stabilizing that designs incorporating non-binding peptides could still be expressed. Crystallographic studies were initially hampered by the low rate of crystallizing human SCTs alone. We evaluated several alternate strategies, including use of the Fab fragment of the anti-pan-HLA-I antibody W6/32 (9, 10) as a co-crystallization chaperone. [We also report the sequence and recombinant expression of W6/32 (**Supplementary Information**).] Success was ultimately achieved by isolating and recombinantly expressing the VHH domain from a novel, high-affinity, anti-β_2_m camelid antibody (11), allowing the crystallization of all SCTs tested.

## 2 Results

### 2.1 SCT production and evaluation

SCTs were engineered based on the general template N–[*leader peptide*]–[*antigenic peptide*]–[*(G_4_S)_3_ linker*]–[*β_2_m*]–[*(G_4_S)_3_ linker*]–[*HLA heavy chain*]–[*6 His tag*]–C and were expressed in HEK293F cells *via* the Daedalus lentiviral transduction system (**Supplementary Information**) (12). We evaluated the following SCT platforms based on the native HLA ectodomain with incorporated combinations of mutations: SCT^Y84A^ which contains the groove-opening mutation Y84A facilitating linker clearance from the groove (8, 13); SCT^H74L/Y84C^ which contains both a stabilizing disulfide between residue 84 in the groove and a cysteine in the peptide/β_2_m linker (GCGGS(G_4_S)_3_) (14, 15) and the stabilizing H74L mutation (16, 17); and SCT^Y84C/A139C^ which contains a stabilizing engineered disulfide at the C-terminal end of the peptide binding cleft between the α1 and α2 helix (18). We also tested novel human A*24:02 α1/α2–murine H-2K^d^ α3/β_2_m chimeric SCTs (χSCT) with the goal of focusing murine humoral responses to improve recovery of TCR-mimic monoclonal antibodies (19) during immunization. For χSCTs, mutations were introduced in the human α-chain to maintain interdomain contacts: K3R, P33S, P34D, and M54L. Lastly, SCT constructs not containing mutations, or only the H74L mutation, were made to assess the contribution of single mutations to stability. Wild type pHLA-I^RF^s were produced using conventional RF protocols (20). 57 different peptides and ten different classical and non-classical HLA alleles, including two χSCTs, were tested: A*01:01, HLA-A*02:01, HLA-A*02:01/K^d^, A*11:01, A*23:01 (pHLA-I^RF^ only), A*24:02, A*24:02/K^d^, B*07:02, B*40:01, C*07:01 (pHLA-I^RF^ only), E*01:03, and G*01:02) (**Supplementary Table 1**). Ten peptides were tested across the full set of seven SCT mutation combinations in HLA-A*02:01-based SCTs (**Supplementary Table 1**), including the classic Wilm’s Tumor protein 1 (WT1) 9-mer epitope RMFPNAPYL (21) and a 9-mer epitope from the E7 oncoprotein of human papillomavirus 16 (HPV16), YMLDLQPET (22). Since we had previously observed presentation of a 12-mer, YMLDLQPETTDL encompassing this 9-mer (2), we included a nested series of 8-mer to 14-mer peptides (YMLDLQPE|T|T|D|L|Y|C, the “YML series”) across this HPV16 E7 sequence in HLA-A*02:01-based pHLA-I^RF^ and SCT constructs. We found that all HLA alleles tested could be expressed as SCTs at <1mg/L to >100 mg/L, depending on presented peptide and SCT mutations.

**Figure 2:**
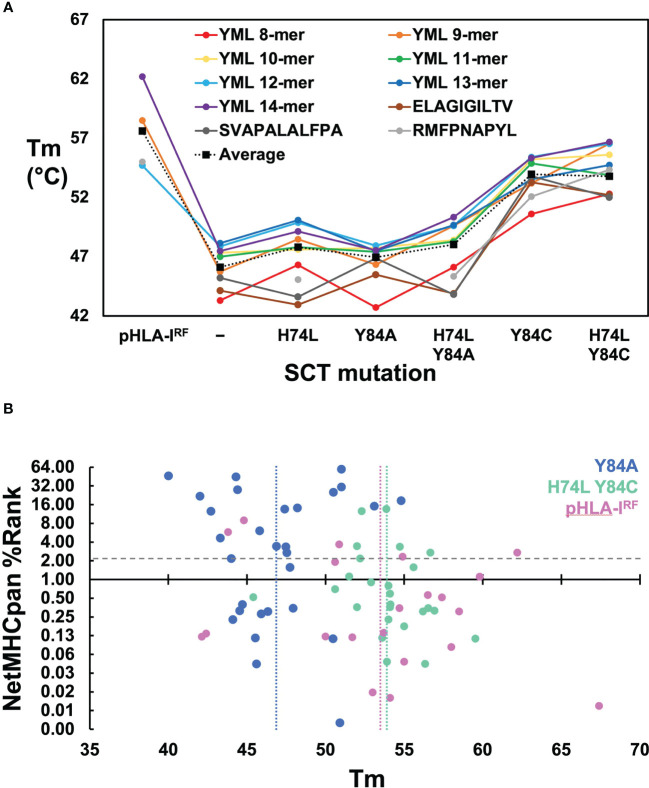
Evaluation of SCT design thermostabilities and comparison of experimental stability with predicted peptide binding ranks. **(A)** Thermostabilities are plotted for a subset of peptide-specific SCTs and peptide-matched pHLA-I^RF^s including averaged thermostabilities for each class of SCT design (Y84: n=14; H74L Y84: n= 14; Y84A: n= 31; H74L Y84A: n= 14; Y84C: n= 18; H74L Y84C: n= 23; Y84C A139C: n= 14) along with the averaged thermostabilities for pHLA-I^RF^s (n=20). Data points shown for peptide-specific SCT designs and matched pHLA-I^RF^s are calculated as an average of three replicate measurements (shown in colored circles). Data points for the “Average” represent an average of all SCTs in the specified SCT class or pHLA-I^RF^ with Tm measurements run in triplicate for each SCT or pHLA-I^RF^ included in the average. **(B)** NetMHCpan predicted binding ranks are plotted versus thermostability measurements for SCTs and pHLA-I^RF^s: Considering pHLA-I^RF^ Tms as the most representative of “native” pHLA-I stability, the measured Tms over 20 examples across six alleles, ranged from 42.1 to 67.4° C, with an average of 53 ± 7° C (*vertical pink line*); the average Tms for Y84A SCT (n=29) and H74L/Y84C (n=25) constructs are 47 ± 3°C (*vertical blue line*) and 54 ± 3°C (*vertical green line*), respectively, noting that this was a very non-random selection of SCTs and pHLA-I^RF^s with few matched peptide/allele pairs.

Solution thermostabilities (Tm) were determined for 149 expressible pHLA-I^RF^s and SCTs by circular dichroism (CD) spectroscopy or fluorescent dye binding. Comparing Tms across pHLA-I^RF^s and SCT constructs with the same peptide provided estimates of the stabilizing effects of different designs; comparing Tms between pHLA-I^RF^s or the same SCT design with different peptides provided surrogate measurements of peptide binding quality. Observed Tm values ranged from 39.4° to over 67° (**Supplementary Table 1**) clearly ranking peptides in terms of relative binding quality and distinguishing between effects of SCT mutations. Compared over HLA-A*02:01-based pHLA-I^RF^s and SCT constructs with common peptides (**Figure 2A**), SCT designs lacking stabilizing disulfides showed an average reduction of ~10° C versus pHLA-I^RF^s. Introducing disulfides into SCTs increased thermostability by an average of ~6° C. The H74L mutation increased thermostability by an average of ~2° C in SCT designs lacking engineered disulfides. While the H74L mutation was only moderately stabilizing, the effect was sufficient to rescue expression of the relatively weakly-binding HLA-A*02:01–RMFPNAPYL SCT(23), which could not be expressed as a simple Y84A SCT but could be produced as a pHLA-I^RF^. Mutations Y84A and H74L had a peptide-dependent effect on stability (**Figure 2A**).

Binding quality predictions were performed for pHLA-I^RF^s and SCTs using the NetMHCpan 4.1 webserver which were then experimentally evaluated for expression yield. There was little correlation between NetMHCpan predicted binding behavior, SCT expression yield, and Tm (**Supplementary Table 1**; **Figure 2B**). Three predicted strong-binding peptide/HLA allele complexes could not be expressed at appreciable levels (prediction false positives) and seven predicted non-binding peptide/allele combinations appreciably expressed, most at very high levels, yielding crystal structures in some cases (*see below*), confirming normal binding (prediction false negatives). Tm measurements were compared for pHLA-I^RF^s and SCTs with NetMHCpan predicted binding ranks (**Figure 2B**), displaying essentially no correlation. Higher Tms did not trend with lower prediction rank, as would be expected (predicted binders score < 2); the peptide with the highest Tm had a non-binding prediction score. SCT expression yield was not correlated with Tm, with the four yield bins from **Supplementary Table 1** showing similar average Tms with large standard deviations (<1 mg/L: 49 ± 5° C; 1-10 mg/L: 47 ± 3° C; 10-40 mg/L: 50 ± 5° C; and >40 mg/L: 51 ± 3° C).

### 2.2 SCT crystallization

Conditions were screened to grow diffraction-quality crystals to enable crystallographic structure determinations for four different SCT designs: Y84A, Y84A χ, H74L Y84C, and Y84C/A139C. Many SCTs failed to crystallize after exhaustive screening so we turned to the anti-pan-HLA antibody W6/32 as a co-crystallization chaperone (9). The sequence of W6/32 was determined from the publicly available hybridoma (ATCC #HB-95), subcloned, and expressed *via* Daedalus in HEK293F cells. The sequencing was confirmed by comparing the binding of recombinant and commercially-sourced W6/32 to A*02:01–YMLDLQPETTDL SCTs by surface plasmon resonance (SPR), which showed identical kinetics (**Supplementary Figure 1A**), confirming successful sequencing and expression. W6/32 Fab was prepared by proteolytic digestion and complexed with SCTs but yielded no usable crystals. In order to obtain a better antibody-based co-crystallization chaperone, a llama was immunized with an SCT^Y84A^. Members of the family Camelidae have antibodies devoid of light chains, and whose antigen recognition site lies exclusively in the heavy chain variable domain (VHH). Subsequent screening of a VHH expression library from the llama yielded one set of closely related sequences around the clonotype archetype AD01. VHH-AD01 was successfully produced as a cleavable Siderocalin fusion protein (24) *via* Daedalus. SPR analyses of the binding of VHH-AD01 to two different SCTs (**Supplementary Figure 1B**) showed *K_D_* s better than 150 pM, a dramatic improvement over W6/32. Co-crystallization with VHH-AD01 yielded diffraction-quality crystals of nine previously recalcitrant SCTs of nine screened, supporting structure determinations.

### 2.3 SCT crystallography

We report a total of 11 SCT crystal structures, in five different triclinic, monoclinic, orthorhombic, or tetragonal space groups, with one, two, or 16 complexes in the asymmetric unit (AU), at resolutions ranging from 1.99 to 2.78 Å (**Supplementary Tables 2**, **3**). While reasonable statistics for indexing and scaling in tetragonal lattices closely related to the triclinic supergroup could be obtained, molecular replacement solutions that adequately packed the AU could only be obtained when reindexed with a monoclinic lattice. However, these solutions ultimately proved incorrect when select chains overlapped and clashed with symmetry mates in these packings. These conflicts could only be overcome in *P*1. Electron density was cleanly interpretable for β_2_m and the α1 and α2 domains of the SCTs, including regions with engineered mutations. However, the α3 domain extended into a solvent channel without crystal contacts resulting in high B-factors and diffuse electron density; portions of the α3 domain in which residues could not be built with a high degree of certainty were left out of the final model. Electron density was not present for the polyglycine-serine linkers save for one to three residues C-terminal of the bound peptide in some complexes.

### 2.4 VHH/β_2_m interface

**Figure 3:**
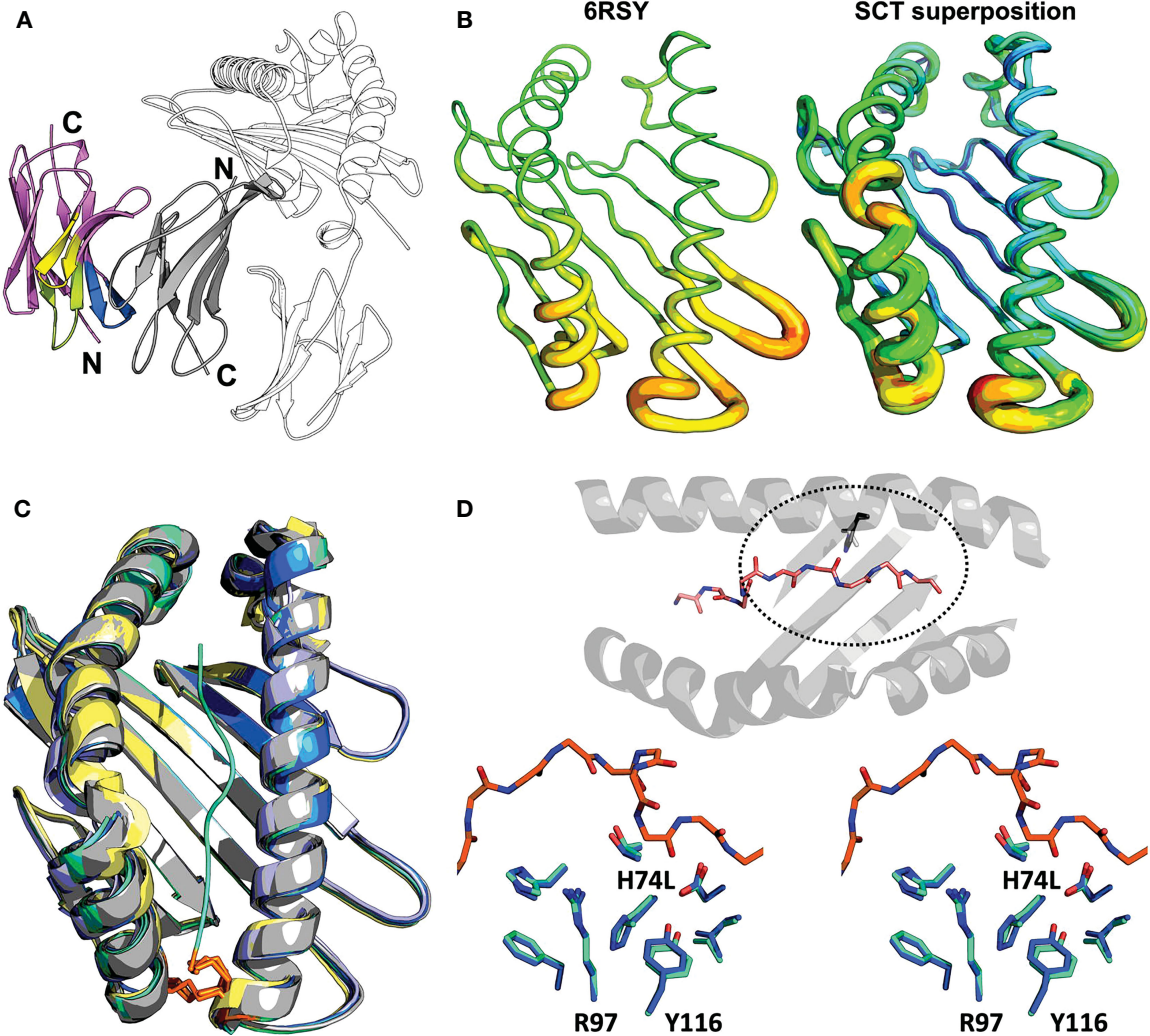
Structural evaluation of SCT designs including the VHH co-crystallization reagent VHH-AD01 **(A)** Recognition of β_2_m by anti-HLA-I VHH-AD01 is shown in cartoon ribbon representation, with the VHH colored *pink* overall with CDR1 highlighted in *lime*, CDR2 in *yellow*, and CDR3 in *blue*. The SCT heavy chain is shown in *black-and-white* cartoon representation with β_2_m in solid *gray* (PDB accession code 7SQP). Secondary structure elements are indicated. **(B)** The native structure of HLA-A*02:01 (PDB accession code 6RSY) is shown on the left with the superposition of all 11 SCTs determined herein shown on the right. Structures are shown in putty representations where the backbone is displayed as a tube with a diameter and color correlating with the experimentally determined B-factors (*blue*/narrow = low B-factor, *red*/fat = high B-factor) on a relative scale. **(C)** Superposition of all HLA-A*02:01 SCT structures reported in this work plus a reference native HLA-I structure are shown in cartoon ribbon representations: SCT^Y84A^ in *grey*; SCT^H74L/Y84C^ in *teal*; SCT^Y84C/A139C^ in *blue*; χSCT in *lavender*; native HLA-A*02:01 (PDB accession code 6RSY) in *yellow*. The YML 14mer peptide from SCT^H74L/Y84C^ (PDB accession code 7SR4) is shown as a *teal* noodle. The side-chains of the Y84C and A139C mutations are shown in *orange* stick representations. Illustrative RMSD values from global alignments performed with PyMOL (25) on all main chain heavy atoms in the α1α2 domains are: 0.19 Å between SCT^Y84A^ (PDB accession code 7SQP) and SCT^H74L/Y84C^ (PDB accession code 7SR3), and 0.18 Å between SCT^H74L/Y84C^ (PDB accession code 7SR4) and SCT^Y84C/A139C^ (PDB accession code 7ST3). For comparison, the RMSD for two identical molecules in the AU of PDB accession code 7ST3 is 0.11 Å, and the RMSD between the reference HLA-A*02:01 structure and SCT^Y84C/A139C^ (PDB accession code 7SR5) with the same WT-1 peptide is 0.45 **(Å)** Pairing of murine H-2K^d^ α3 with human HLA-A*02:01 α1/α2 domains in the murine/human χSCT also had little effect on the overall structure of the binding cleft, with an alignment yielding an RMSD of 0.24 Å between SCT^Y84A χ^ (PDB accession code 6E1I) and SCT^Y84A^ (PDB accession code 7SQP). **(D)** *Top*: bird’s eye view of the α1α2 domain, shown as a cartoon ribbon, showing the bound peptide in a stick representation colored by atom type and the side-chain of residue 74, H or L, circled with a dashed line. *Bottom*: stereo view of the side-chains of residues neighboring the H74L mutation from the superposition of the YML12-mer SCT^H74L/Y84C^ (*teal*; PDB accession code 7SR3) and YML12-mer SCT^Y84A^ (*blue*; PDB accession code 7SQP) structures. The main chain of the YML 12-mer peptide is shown with carbon atoms in *orange* in both views for reference.

Clearly interpretable electron density was apparent for the entire VHH-AD01 moiety in the complex showing VHH-AD01 bound without molecular contacts to the HLA heavy chain, linker, or HLA-bound peptide; therefore, recognition of the HLA complex is neither construct nor allele specific. The binding mode of VHH-AD01 to β_2_m is unlike conventional antibody variable domain interactions, predominately *via* the complementary determining region (CDR) loops. Instead, VHH-AD01 recognizes β_2_m with a side-to-side orientation (**Figure 3A**), with VHH CDR regions extending edgewise onto the interacting β-sheet. This binding orientation buried the surface normally in contact with the V_L_ domain in conventional antibodies with paired V_H_/V_L_ cassettes – but in a head-to-tail, not head-to-head orientation. VHH-AD01 residues 37 through 47, which comprise a large portion of a conventional V_H_/V_L_ interface (e.g., 264 Å^2^ in 4LRN.pdb (24)), also comprise a large portion of the VHH-AD01/β_2_m interface (e.g., 308 Å^2^ in 7SQP). Approximately one quarter of all surface exposed residues in each domain are involved in the binding interface with a total buried surface area of 874 Å^2^ including eight salt bridges and 17 hydrogen bonds (**Supplementary Table 4**).

### 2.5 Effect of SCT design variation on HLA structure

We found the overall structure of the α1α2 peptide-binding platform was minimally affected by SCT mutations or by chimeric pairing (**Figures 3B, C**). Structures with or without the H74L mutation showed that surrounding residues in the A and F binding pockets were nearly identical (**Figure 3D**) However, the buried surface area of leucine increased by 6.5 Å^2^ over histidine showing a more favorable packing. The change in enthalpy when introducing the H74L mutation was found to be favorable with a ΔΔG of 1.9 Kcal/mol as calculated by DDGun (26). This modest change could translate to an increase in overall stability, but the peptide dependence of this gain cannot readily be explained by our structures.

### 2.6 Effect of SCT design on peptide presentation

**Figure 4:**
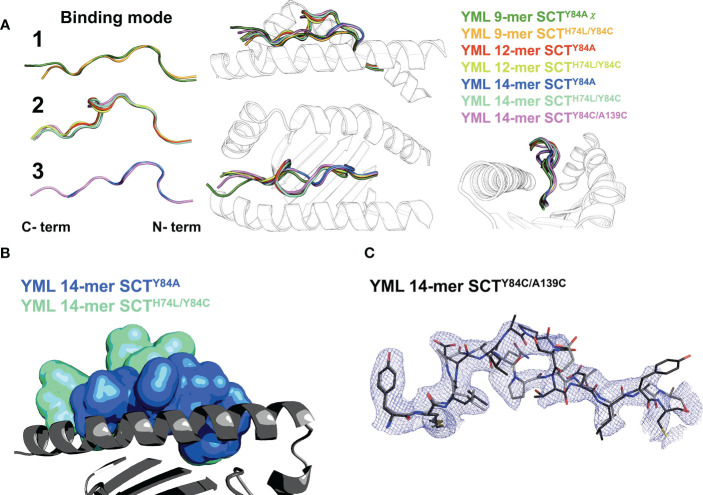
Peptide binding in different SCT designs. **(A)** *Left*: peptide binding modes observed across the YML series, with peptides shown as main-chain backbones in cartoon representations, colored according to peptide length and SCT design as indicated. *Right*: three orthogonal views of the superposition of all peptides are shown with the α1α2 domain shown as a *black-and-white* cartoon ribbon. **(B)** The YML 14-mer peptide is shown in two different SCT design-dependent conformations in a superposition of the SCT^Y84A^ and SCT^H74L/Y84C^ structures. The peptides are shown in molecular surface representations colored as indicated. The α1α2 domain, colored *dark gray* with the α2 helix removed for clarity, is shown as a cartoon ribbon oriented with the peptide C-terminus on the left. **(C)** The electron density map (*blue* mesh) is shown for the YML 14-mer SCT^Y84C/A139C^ structure, illustrating the two alternative binding modes present in the structure. The YML 14-mer peptides are shown in stick representations, oriented with N-termini on the left, colored with carbon atoms either in *black* or *dark gray*.

While not affecting the HLA-I fold, the choice of SCT design could have a large effect on peptide presentation. Difference Fourier OMIT maps (**Supplementary Figure 2**) (27, 28) clearly show specific peptide binding in all the cognate complexes. Canonical presentation was observed for the 9-mer peptides, with residues p2 and p9 occupying the primary anchors in the B- and F-pockets, respectively, regardless of the SCT design background (YML 9-mers shown in canonical “binding mode 1”; **Figure 4A**). The YML 12-mer peptide loops outward over the central binding pocket, typical of longer peptides (e.g., (29)), with residues p2 and p12 anchoring the peptide across SCT designs (“binding mode 2”; **Figure 4A**).

The YML 14-mer peptide was found in two different, out-of-register conformations using two different C-terminal anchors depending on SCT design (**Figure 4B**). When Y84A is introduced, the 14-mer binds with residues p2 and p10 as anchors (“binding mode 3”; **Figures 4A, B**). The remaining C-terminal residues extended out of the groove through the now-unconstrained end. In contrast, when a disulfide is introduced between the second residue in the peptide linker and Y84C, the YML 14-mer binds identically to the YML 12-mer peptide, with p2 and p12 occupying the primary anchors in the canonical register, using binding mode 2 (**Figures 4A, B**). The disulfide did not form between the introduced cysteines in the linker and residue 84, but between the cysteine at position 14 of the YML peptide and residue 84. Formation of the unintended disulfide occurred in both complexes in the AU. The YML 14-mer peptide was found in both binding mode 2 and 3 in the SCT^Y84C/A139C^ background. The electron density clearly showed this peptide in both states (**Figure 4C**) indicating that either the peptide loaded stochastically sampling both conformers or was able to reequlibrate after preferential loading in one.

### 2.7 8-mer binding mode of HLA-A*24

**Figure 5:**
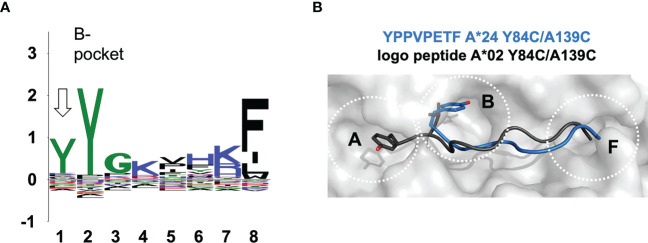
The observed noncanonical 8-mer binding mode. **(A)** Sequence logo generated from 1,883 8-mer peptides isolated from HLA-A*24:02 *via* the Artemis mass-spec procedure^2^ is shown. The p1-p8 peptide positions are graphed on the abscissa and information content at each position in bits is graphed on the ordinate. Note that the tyrosine constituting the p2 anchor residue for HLA-A*24:02 9-mer and longer peptides partially shifts register to p1 for 8-mers (*arrow*), indicating utilization of an alternate binding mode where 8-mer p1 occupies the B-pocket. **(B)** The 8-mer YPPVPETF peptide/SCT crystal structure (tyrosine at p1; PDB accession code 7SRK) is shown as a *gray* molecular surface, with the peptide shown in blue in a main-chain cartoon/side-chain stick representation, binding in the non-canonical mode. The logo peptide (YLAAAAAAV, tyrosine at p2) from the A*02:01 SCT^Y84C/A139C^ crystal structure (PDB accession code 7STG) is superimposed in *black* to contrast canonical binding.

Using our ARTEMIS platform for determination of HLA-restricted peptides, we determined a length-specific peptide logo for HLA-A*24:02 computed from 1,883 8-mer peptides (**Figure 5A**)^2^. We found, along with the canonical binding mode (p2 occupying the B-pocket), an alternative binding mode for 8-mer peptides where position p1 echoed the amino acid preference of the B-pocket. This logo pattern would arise if a subset of 8-mer peptides did not completely fill the peptide-binding groove, leaving the A-pocket empty and filling the B-pocket with the side chain of p1, as had been observed in one prior pHLA-I structure: 1DUY.pdb (30). To confirm this, we expressed an ARTEMIS-identified self-peptide from the RADX protein, YPPVPETF, in an A*24:02 SCT^Y84C/A139C^ background. The 2.5 Å resolution structure, with two complexes in the AU, showed clear electron density in the groove confirming that this 8-mer peptide bound leaving the A-pocket empty, with p1 (tyrosine) filling the B-pocket (**Figure 5B**; **Supplementary Figure 2**). Residues at p2, p4, p5/6, and p8, bound in the D, C, E, and F-pockets, respectively, recapitulating the canonical binding mode for an A24 8-mer peptide save for residues P5 and P6, which extend slightly out of the groove, each partially filling the E-pocket and taking up slack for the shifted register.

### 2.8 Presentation of endogenous peptides by SCT^Y84C/A139C^

Since the Y84C/A139C mutation had been reported to yield “empty” HLA-Is by *iv*RF in the absence of specific introduced peptides (31, 32), we tested a low affinity peptide incorporated into the SCT design; the A*24-associated YPPVPETF peptide in the A*02:01 SCT^Y84C/A139C^. The 2.7 Å resolution structure showed clear peptide density inconsistent with the YPPVPETF peptide in all 16 complexes in the AU (**Supplementary Figure 2**). Density features in the B-pocket were inconstant with either tyrosine (p1 in non-canonical mode) or proline (p2 in canonical mode). A 9-mer “logo” peptide with A*02:01 consensus sequence anchors (YLAAAAAAV) provided a much-improved fit and resulted in improved R_free_ during refinement over the linked YPPVPETF peptide (**Figure 5B**; **Supplementary Figure 2**) but was not included in the deposited coordinates, as the density features are best described as an averaged ensemble of bound peptides. This result demonstrated that disulfides introduced into SCT designs can be so stabilizing as to overcome inadequate linked peptide binding quality as an expression requirement.

## 3 Discussion

The engineered SCT format has mixed advantages and limitations as a surrogate for native pHLAs in biochemical studies. The key advantage is permitting expression in eukaryotic systems, where the key caveats are destabilization, which can foil expression of weakly binding peptides, or over-stabilization by point mutations and engineered disulfide linkages, leading to decoupling of linked peptide binding from native peptide presentation. Another practical SCT disadvantage is reducing crystallizability, overcome by our isolation of a dependable VHH crystallization chaperone. Our reported structural studies using this approach showed overall conservation of native peptide recognition by SCTs across most examples analyzed, with the important caveat that long peptides could adopt multiple or non-canonical binding modes due to register shifts in less-constrained SCTs. These studies also reconfirmed non-canonical binding by a subset of 8-mer peptides, an important consideration for TCR recognition. Considering *in vitro* refolded, soluble versions of pHLAs as the reference standards, biochemical assessments of Tms (as a surrogate metric of peptide binding quality) showed a wide range across different peptides, with clear destabilization in the SCT formats not including stabilizing mutations. These analyses also failed to show meaningful correlations between computational predictions of binding quality, expressibility, and experimental Tm measurements. The take-home lesson is that previously unappreciated complications in the SCT format and unreliability of readily available predictions can confound the applicability of these approaches unless careful consideration is applied. We suggest the following general guidelines for choosing an appropriate SCT design, based on characteristics of the linked peptide: SCT^Y84C/A139C^ should be avoided due to over-stabilization resulting in expression of any SCT, regardless of whether peptide is bound. The Y84C mutation should be avoided for cysteine-containing peptides due to unintended disulfide formation between the peptide and heavy chain resulting in off register binding; use Y84A instead. Verification of binding register is needed when designing SCTs with peptides longer that 10 residues, especially when peptides contain alternate anchor positions. SCT^H74L/Y84C^ should be used when incorporating low affinity peptides to ensure expression; H74L alone can rescue expression of some SCTs with low affinity peptides.

## 4 Materials and methods

### 4.1 Protein expression and purification

General sequences are given for all protein constructs in the **Supplementary Information**. cDNAs encoding SCTs and other proteins were codon optimized for human cells (Genscript), synthesized (Genscript), and subcloned into optimized lentiviral vectors incorporating fluorescent reporter proteins (12). Transductions and protein expression and purification were carried out as previously described (12): HEK293 Freestyle cells (Invitrogen #R79007, RRID: CVCL_D6642) were grown in Freestyle 293 Expression media (Gibco #12338018) with shaking at 130 rpm, 37° C, 8% CO_2_ in vented shake flasks. Cells were transduced, with near 100% efficiencies as judged by reporter fluorescence, at a density of 10^6^ cells/mL in 10 mL Freestyle media. Cultures were grown to ~200 mL at which point reduced/non-reduced SDS PAGE gels were run with 20 mL 10x supernatant to determine expression levels and confirm proper folding (**Supplementary Figure 3**). Cultures were harvested once densities reached ~8 x 10^6^ cells/mL in 4 L total culture volume. Cells were pelleted and supernatant was 0.2 micron filtered and supplemented with 150 mM NaCl before purification using Ni-NTA agarose (Qiagen #30210). Columns were washed with buffers containing 10 mM imidazole before elution with buffers containing 250 mM imidazole. Eluted protein was further purified by preparative SEC on a Superdex 75 column (GE Health Sciences) in 10 mM PIPES, pH 7.1, 150 mM NaCl, 1 mM EDTA. pHLA-I^RF^s were produced as monomers through the FHCC Tetramer Facility as described (33).

### 4.2 VHH isolation

Two llamas were immunized on weeks 0, 2, 4, 8 and 12 with 500 µg of HLA-A*0201/YMLDLQPETTDL SCT^Y84A^ formulated with Fama 3030 adjuvant (GERBU Biotechnik). On weeks 10 and 14, 500 ml of whole blood were drawn from each animal and PBMCs were isolated by Ficoll gradient and washed with PBS. RNA was isolated from 1 x 10^7^ cells using RNA isolation kits (Qiagen #74106) following the manufacturer’s protocols. cDNA was generated using a SuperScript IV reverse transcriptase kit (ThermoFisher #18090010) following the manufacturer’s protocols. The primer used for amplification was AL.CH2.2 (34). VHHs were amplified from bulk cDNA by PCR with two sets of primers: VH1 BACK was used as forward primer in both reactions (35); Lam08 and AlpVHH R1 were used as the reverse primers to amplify IgG2 and IgG3 isotypes, respectively (34). The primers contained overlap regions with the yeast display plasmid to allow recombination with the vector after electroporation.

For generating a yeast surface-display library, VHH fragments amplified from llama cDNA and linearized pETcon vector (RRID : Addgene_41522) were electroporated as previously described (36) into *Saccharomyces cerevisiae* strain EBY100 (ATCC #MYA-4941(37)). Aliquots of the library (5 x 10^9^ cells) were then thawed, washed, induced, washed, and stained with 10 µg/ml of AlexaFluor 488-conjugated anti-cMyc antibody (clone 9E10, Biolegend #626812) in PBS, 2% FBS, 1 mM EDTA. The aliquot was subsequently washed and incubated for 30 minutes with anti-Fc magnetic beads and then enriched for Myc-expressing cells by passing through an AutoMacs magnetic separator. Subsequent rounds of selection with enriched library aliquots were performed with a Becton Dickinson Aria II cell sorter, isolating cells double positive for Myc expression and for binding to biotinylated SCT antigen coupled to fluorescently labeled streptavidin. The concentration of antigen was decreased for each round of selection, from 1 µM down to 1 nM. Yeast showing selectivity for antigen at 1 nM were single cell sorted into 96-well plates for clonal expansion.

### 4.3 Surface plasmon resonance

Surface plasmon resonance (SPR) experiments were performed at 25°C on a Biacore T100 instrument (Cytiva). VHH-AD01 binding experiments were run with a Series S SA chip and 10 mM HEPES, pH 7.4, 150 mM NaCl, 3 mM EDTA, 0.05% Tween 20 (HBS-EP+) with 0.1 mg/mL bovine serum albumin (BSA) as the running buffer. Each biotinylated SCT was injected at 10 mL/minute to capture ~120 RUs; a blank streptavidin flow cell was used for referencing. VHH-AD01 binding was performed using a kinetic titration method. Four buffer blank cycles were run prior to the cycle with all but the first averaged for double-referencing. VHH-AD01 was injected sequentially at 0.56, 1.6, 5, 15, and 45 nM at 50 mL/minute for 7 minutes with a final dissociation of 2 hours. Data were analyzed using a single cycle kinetic analysis model in BiaEvaluation 2.0.4 software. Figures were made in Prism 9 for Mac OS software.

W6/32 experiments were run with goat anti-mouse IgG, Fcγ fragment specific antibody (Jackson ImmunoResearch #115-005-071) amine coupled to 4 flow cells of a Series S CM5 chip (~7400 RUs). The running buffer was HBS-EP+ with BSA added after amine coupling. W6/32 was injected at 0.5 mg/mL, 10 mL/minute to capture 85 RUs of RKS-400/401 (W6/32 with mouse Fc) or 82 RUs of commercial W6/32 (BioLegend #311402). A buffer blank and single concentration of SCT (10 mM) were injected at 50 mL/minute for seven minutes over both the captured W6/32 constructs and two anti-mouse IgG Fcγ alone surfaces, then allowed to dissociate for 20 minutes. Data was double-referenced then normalized by dividing each curve by its maximum response in Scrubber 2.0c software (BioLogic Software). Maximum binding responses observed were 37.8 and 13.4 RUs for in-house and BioLegend W6/32, respectively. The overlay plot was made in Prism 9 for Mac OS software.

### 4.4 Crystallization and structure determination

All SCT/VHH scaffold complexes were isolated by SEC, concentrated to ~10 mg/ml, and crystallized by vapor diffusion at room temperature. Reservoir solutions are given for each crystal in **Supplementary Table 2**. Crystals were cryo-protected by transferring into well solutions containing 15-20% glycerol and kept at −170°C during diffraction data collection. Diffraction data for 7SQP.pdb, 7SR0.pdb, 7SR3.pdb, 7SR4.pdb, 7SSH.pdb, 7ST3.pdb, 7SR5.pdb, and 7STG.pdb were collected at the Advanced Light Source (Berkeley, CA) beamline 5.0.1/5.0.2 and integrated and scaled with HKL-2000. Diffraction data for 6APN.pdb and 6E1I.pdb were collected in house with CuKα radiation on an R-AXIS IV++ image plate detector with HR optics (Rigaku) and integrated and scaled with HKL-2000. Diffraction data for 7SRK.pdb were collected in house with CuKα radiation on an XtaLab Synergy diffractometer with HyPix-6000HE detector (Rigaku) and integrated and scaled with Rigaku CrysAlisPro. Initial phases were determined by molecular replacement using Phaser as implemented in the CCP4 software suite (38, 39), using coordinate set 1JF1.pdb (40) as a model of the HLA and 1I3V.pdb (41) as a model for the VHH. Phases were improved by subsequent rounds of model building and refinement using COOT (42) and REFMAC (43) or Phenix (44, 45). Polder OMIT maps (27, 28) were calculated in Phenix. Structure validation was carried out with the MolProbity server (46–48), and the RCSB PDB ADIT validation server (49). Data collection and structure refinement statistics are shown in **Supplementary Table 3**. Deposited structures are numbered continuously from 1 to the final residue in the SCT to comply with PDB conventions, but are discussed in this work as numbered from 1 to the end of each chain separately, excluding linkers. The CDRs for AD01 VHH were predicted by PyIgClassify (50) and residues are numbered following the North-AHO numbering scheme (51). VHH/β_2_m interfaces were calculated with PDBePISA (52).

### 4.5 Tm analyses

Thermal denaturation measurements by fluorescent thermal shift-based assays were performed in triplicate using ~10 mg of protein plus a five-fold molar excess of SYPRO Orange (Invitrogen S6651) in a total volume of 20 μL in PBS. Melting temperature measurements were obtained using a Bio-Rad CFX96 real-time PCR instrument with a HEX filter. Temperature was continuously increased 2 °C/min from 25°C to 95°C. Tms were estimated from the inflection point of the fluorescence response curve. Alternately, thermal denaturation measurements by CD were obtained using 15 mmol/L protein in 10 mM KPO4, pH 7.0, total volume 300 μL on a Jasco J-815 spectrometer with Jasco Peltier temperature controller PFD-4256. Temperature was increased from 20 to 95°C with measurements collected every 0.5°C at a wavelength of 220 nM and path length of 1 mm. Tms were estimated from the inflection point of the CD response curve.

## Data availability statement

Crystallographic structure factors and model coordinates have been deposited in the Protein Data Bank under accession codes: 7SQP, 7SR0, 7SR3, 7SR4, 7SR5, 7SRK, 7SSH, 7ST3, 7STG, 6APN, and 6E1I.

## Author contributions

KF, PR, WC, JH, and RS designed the study, analyzed results, and wrote the manuscript; KF, PR, DF, MB, UF-M, and DR performed the biochemical studies; and EL, JC, and RP isolated the VHH. All authors contributed to the article and approved the submitted version.

## References

[1] Rock KL Reits E Neefjes J. Present yourself! by MHC class I and MHC class II molecules. Trends Immunol (2016) 37:724–37. doi: 10.1016/j.it.2016.08.010 PMC5159193 27614798

[2] Finton K Brusniak M-Y Jones LA Lin C Fioré-Gartland AJ Brock C. ARTEMIS: a novel mass-spec platform for HLA-restricted self and disease-associated peptide discovery. Front Immunol (2021) 12:658372. doi: 10.3389/fimmu.2021.658372 33986749 PMC8111693

[3] Rudolph MG Stanfield RL Wilson IA. How TCRs bind MHCs, peptides, and coreceptors. Annu Rev Immunol (2006) 24:419–66. doi: 10.1146/annurev.immunol.23.021704.115658 16551255

[4] Finton KA Strong RK. Structural insights into activation of antiviral NK cell responses. Immunol Rev (2012) 250:239–57. doi: 10.1111/j.1600-065X.2012.01168.x PMC3471384 23046134

[5] Strong RK. Structural immunology of MHC class I proteins, homologs and receptor complexes. Modern Aspects Immunobiol (2000) 3:125–8.

[6] Hansen TH Connolly JM Gould KG Fremont DH. Basic and translational applications of engineered MHC class I proteins. Trends Immunol (2010) 31:363–9. doi: 10.1016/j.it.2010.07.003 PMC2949479 20832361

[7] Strong RK Holmes MA Li P Braun L Lee N Geraghty DE. HLA-e allelic variants. correlating differential expression, peptide affinities, crystal structures, and thermal stabilities. J Biol Chem (2003) 278:5082–90. doi: 10.1074/jbc.M208268200 12411439

[8] Yu YY Netuschil N Lybarger L Connolly JM Hansen TH. Cutting edge: single-chain trimers of MHC class I molecules form stable structures that potently stimulate antigen-specific T cells and b cells. J Immunol (2002) 168:3145–9. doi: 10.4049/jimmunol.168.7.3145 11907065

[9] Barnstable CJ Bodmer WF Brown G Galfre G Milstein C Williams AF. Production of monoclonal antibodies to group a erythrocytes, HLA and other human cell surface antigens-new tools for genetic analysis. Cell (1978) 14:9–20. doi: 10.1016/0092-8674(78)90296-9 667938

[10] Parham P Barnstable CJ Bodmer WF. Use of a monoclonal antibody (W6/32) in structural studies of HLA-A,B,C antigens. J Immunol (1979) 123:342–9. doi: 10.4049/jimmunol.123.1.342 87477

[11] Muyldermans S. Nanobodies: natural single-domain antibodies. Annu Rev Biochem (2013) 82:775–97. doi: 10.1146/annurev-biochem-063011-092449 23495938

[12] Bandaranayake AD Correnti C Ryu BY Brault M Strong RK Rawlings DJ. Daedalus: A robust, turnkey platform for rapid production of decigram quantities of active recombinant proteins in human cell lines using novel lentiviral vectors. Nucleic Acids Res (2011) 39:e143. doi: 10.1093/nar/gkr706 21911364 PMC3241668

[13] Lybarger L Yu YY Miley MJ Fremont DH Myers N Primeau T. Enhanced immune presentation of a single-chain major histocompatibility complex class I molecule engineered to optimize linkage of a c-terminally extended peptide. J Biol Chem (2003) 278:27105–11. doi: 10.1074/jbc.M303716200 12732632

[14] Mitaksov V Truscott SM Lybarger L Connolly JM Hansen TH Fremont DH. Structural engineering of pMHC reagents for T cell vaccines and diagnostics. Chem Biol (2007) 14:909–22. doi: 10.1016/j.chembiol.2007.07.010 PMC3601489 17719490

[15] Truscott SM Lybarger L Martinko JM Mitaksov VE Kranz DM Connolly JM. Disulfide bond engineering to trap peptides in the MHC class I binding groove. J Immunol (2007) 178:6280–9. doi: 10.4049/jimmunol.178.10.6280 17475856

[16] Tussey LG Matsui M Rowland-Jones S Warburton R Frelinger JA Mcmichael A. Analysis of mutant HLA-A2 molecules. differential effects on peptide binding and CTL recognition. J Immunol (1994) 152:1213–21. doi: 10.4049/jimmunol.152.3.1213 8301126

[17] Matsui M Kawano M Matsushita S Akatsuka T. Introduction of a point mutation into an HLA class I single-chain trimer induces enhancement of CTL priming and antitumor immunity. Mol Ther Methods Clin Dev (2014) 1:14027. doi: 10.1038/mtm.2014.27 26015969 PMC4362367

[18] Hein Z Uchtenhagen H Abualrous ET Saini SK Janssen L Van Hateren A. Peptide-independent stabilization of MHC class I molecules breaches cellular quality control. J Cell Sci (2014) 127:2885–97. doi: 10.1242/jcs.145334 24806963

[19] Dubrovsky L Dao T Gejman RS Brea EJ Chang AY Oh CY. T Cell receptor mimic antibodies for cancer therapy. Oncoimmunology (2016) 5:e1049803. doi: 10.1080/2162402X.2015.1049803 26942058 PMC4760335

[20] O’callaghan CA Tormo J Willcox BE Blundell CD Jakobsen BK Stuart DI. Production, crystallization, and preliminary x-ray analysis of the human MHC class ib molecule HLA-e. Protein Sci (1998) 7, 1264–1266. doi: 10.1002/pro.5560070525 9605335 PMC2143998

[21] Dao T Yan S Veomett N Pankov D Zhou L Korontsvit T. Targeting the intracellular WT1 oncogene product with a therapeutic human antibody. Sci Trans Med (2013) 5:176ra133–176ra133. doi: 10.1126/scitranslmed.3005661 PMC3963696 23486779

[22] Ressing ME Sette A Brandt RM Ruppert J Wentworth PA Hartman M. Human CTL epitopes encoded by human papillomavirus type 16 E6 and E7 identified through *in vivo* and *in vitro* immunogenicity studies of HLA-A*0201-binding peptides. J Immunol (1995) 154:5934–43. doi: 10.4049/jimmunol.154.11.5934 7538538

[23] Hellman LM Yin L Wang Y Blevins SJ Riley TP Belden OS. Differential scanning fluorimetry based assessments of the thermal and kinetic stability of peptide-MHC complexes. J Immunol Methods (2016) 432:95–101. doi: 10.1016/j.jim.2016.02.016 26906089 PMC4837003

[24] Finton KA Friend D Jaffe J Gewe M Holmes MA Larman HB. Ontogeny of recognition specificity and functionality for the broadly neutralizing anti-HIV antibody 4E10. PloS Pathog (2014) 10:e1004403. doi: 10.1371/journal.ppat.1004403 25254371 PMC4177983

[25] Schrodinger, Llc. The PyMOL molecular graphics system, version 2.0. Schrödinger, LLC. (2010).

[26] Montanucci L Capriotti E Frank Y Ben-Tal N Fariselli P. DDGun: an untrained method for the prediction of protein stability changes upon single and multiple point variations. BMC Bioinf (2019) 20:335. doi: 10.1186/s12859-019-2923-1 PMC6606456 31266447

[27] Bhat TN Cohen GH. OMITMAP: an electron density map suitable for the examination of errors in a macromolecular model. J Appl Crystallogr (1984) 17:244–8. doi: 10.1107/S0021889884011456

[28] Liebschner D Afonine PV Moriarty NW Poon BK Sobolev OV Terwilliger TC. Polder maps: improving OMIT maps by excluding bulk solvent. Acta Crystallogr D Struct Biol (2017) 73:148–57. doi: 10.1107/S2059798316018210 PMC5297918 28177311

[29] Liu YC Miles JJ Neller MA Gostick E Price DA Purcell AW. Highly divergent T-cell receptor binding modes underlie specific recognition of a bulged viral peptide bound to a human leukocyte antigen class I molecule. J Biol Chem (2013) 288:15442–54. doi: 10.1074/jbc.M112.447185 PMC3668706 23569211

[30] Khan AR Baker BM Ghosh P Biddison WE Wiley DC. The structure and stability of an HLA-A*0201/octameric tax peptide complex with an empty conserved peptide-n-terminal binding site. J Immunol (2000) 164:6398–405. doi: 10.4049/jimmunol.164.12.6398 10843695

[31] Moritz A Anjanappa R Wagner C Bunk S Hofmann M Pszolla G. High-throughput peptide-MHC complex generation and kinetic screenings of TCRs with peptide-receptive HLA-A*02:01 molecules. Sci Immunol (2019) 4. doi: 10.1126/sciimmunol.aav0860 31324691

[32] Saini SK Tamhane T Anjanappa R Saikia A Ramskov S Donia M. Empty peptide-receptive MHC class I molecules for efficient detection of antigen-specific T cells. Sci Immunol (2019) 4. doi: 10.1126/sciimmunol.aau9039 31324690

[33] Altman JD Moss PA Goulder PJ Barouch DH Mcheyzer-Williams MG Bell JI. Phenotypic analysis of antigen-specific T lymphocytes. Science (1996) 274:94–6. doi: 10.1126/science.274.5284.94 21690331

[34] Maass DR Sepulveda J Pernthaner A Shoemaker CB. Alpaca (Lama pacos) as a convenient source of recombinant camelid heavy chain antibodies (VHHs). J Immunol Methods (2007) 324:13–25. doi: 10.1016/j.jim.2007.04.008 17568607 PMC2014515

[35] Harmsen MM Ruuls RC Nijman IJ Niewold TA Frenken LG De Geus B. Llama heavy-chain V regions consist of at least four distinct subfamilies revealing novel sequence features. Mol Immunol (2000) 37:579–90. doi: 10.1016/S0161-5890(00)00081-X 11163394

[36] Benatuil L Perez JM Belk J Hsieh CM. An improved yeast transformation method for the generation of very large human antibody libraries. Protein Eng Des Sel (2010) 23:155–9. doi: 10.1093/protein/gzq002 20130105

[37] Chao G Lau WL Hackel BJ Sazinsky SL Lippow SM Wittrup KD. Isolating and engineering human antibodies using yeast surface display. Nat Protoc (2006) 1:755–68. doi: 10.1038/nprot.2006.94 17406305

[38] Potterton E Briggs P Turkenburg M Dodson E. A graphical user interface to the CCP4 program suite. Acta Crystallogr D Biol Crystallogr (2003) 59:1131–7. doi: 10.1107/S0907444903008126 12832755

[39] Winn MD Ballard CC Cowtan KD Dodson EJ Emsley P Evans PR. Overview of the CCP4 suite and current developments. Acta Crystallogr D Biol Crystallogr (2011) 67:235–42. doi: 10.1107/S0907444910045749 PMC3069738 21460441

[40] Sliz P Michielin O Cerottini JC Luescher I Romero P Karplus M. Crystal structures of two closely related but antigenically distinct HLA-A2/melanocyte-melanoma tumor-antigen peptide complexes. J Immunol (2001) 167:3276–84. doi: 10.4049/jimmunol.167.6.3276 11544315

[41] Spinelli S Tegoni M Frenken L Van Vliet C Cambillau C. Lateral recognition of a dye hapten by a llama VHH domain. J Mol Biol (2001) 311:123–9. doi: 10.1006/jmbi.2001.4856 11469862

[42] Emsley P Cowtan K. Coot: model-building tools for molecular graphics. Acta Crystallogr D Biol Crystallogr (2004) 60:2126–32. doi: 10.1107/S0907444904019158 15572765

[43] Murshudov GN Vagin AA Dodson EJ. Refinement of macromolecular structures by the maximum-likelihood method. Acta Crystallogr D Biol Crystallogr (1997) 53:240–55. doi: 10.1107/S0907444996012255 15299926

[44] Adams PD Grosse-Kunstleve RW Hung LW Ioerger TR Mccoy AJ Moriarty NW. PHENIX: building new software for automated crystallographic structure determination. Acta Crystallogr D Biol Crystallogr (2002) 58:1948–54. doi: 10.1107/S0907444902016657 12393927

[45] Liebschner D Afonine PV Baker ML Bunkoczi G Chen VB Croll TI. Macromolecular structure determination using X-rays, neutrons and electrons: recent developments in phenix. Acta Crystallogr D Struct Biol (2019) 75:861–77. doi: 10.1107/S2059798319011471 PMC6778852 31588918

[46] Davis IW Leaver-Fay A Chen VB Block JN Kapral GJ Wang X. MolProbity: all-atom contacts and structure validation for proteins and nucleic acids. Nucleic Acids Res (2007) 35:W375–383. doi: 10.1093/nar/gkm216 PMC1933162 17452350

[47] Chen VB Arendall WB 3rd Headd JJ Keedy DA Immormino RM Kapral GJ. MolProbity: all-atom structure validation for macromolecular crystallography. Acta Crystallogr D Biol Crystallogr (2010) 66:12–21. doi: 10.1107/S0907444909042073 20057044 PMC2803126

[48] Williams CJ Headd JJ Moriarty NW Prisant MG Videau LL Deis LN. MolProbity: more and better reference data for improved all-atom structure validation. Protein Sci (2018) 27:293–315. doi: 10.1002/pro.3330 29067766 PMC5734394

[49] Berman HM Westbrook J Feng Z Gilliland G Bhat TN Weissig H. The protein data bank. Nucleic Acids Res (2000) 28:235–42. doi: 10.1093/nar/28.1.235 PMC102472 10592235

[50] Adolf-Bryfogle J Xu Q North B Lehmann A Dunbrack RL Jr. PyIgClassify: a database of antibody CDR structural classifications. Nucleic Acids Res (2015) 43:D432–438. doi: 10.1093/nar/gku1106 PMC4383924 25392411

[51] Honegger A Pluckthun A. Yet another numbering scheme for immunoglobulin variable domains: an automatic modeling and analysis tool. J Mol Biol (2001) 309:657–70. doi: 10.1006/jmbi.2001.4662 11397087

[52] Krissinel E Henrick K. Inference of macromolecular assemblies from crystalline state. J Mol Biol (2007) 372:774–97. doi: 10.1016/j.jmb.2007.05.022 17681537

