# Supplementary material for: Effects of HLA single chain trimer design on peptide presentation and stability
Source: Front Immunol. 2023 May 3;14:1170462. doi: 10.3389/fimmu.2023.1170462 (PMC10189100; doi:10.3389/fimmu.2023.1170462)
Supplement: Supplementary file 1 [file DataSheet_1.docx]

**Supplementary Figure 1: SPR analyses of anti-β_2_m co-crystallization reagents.**

**
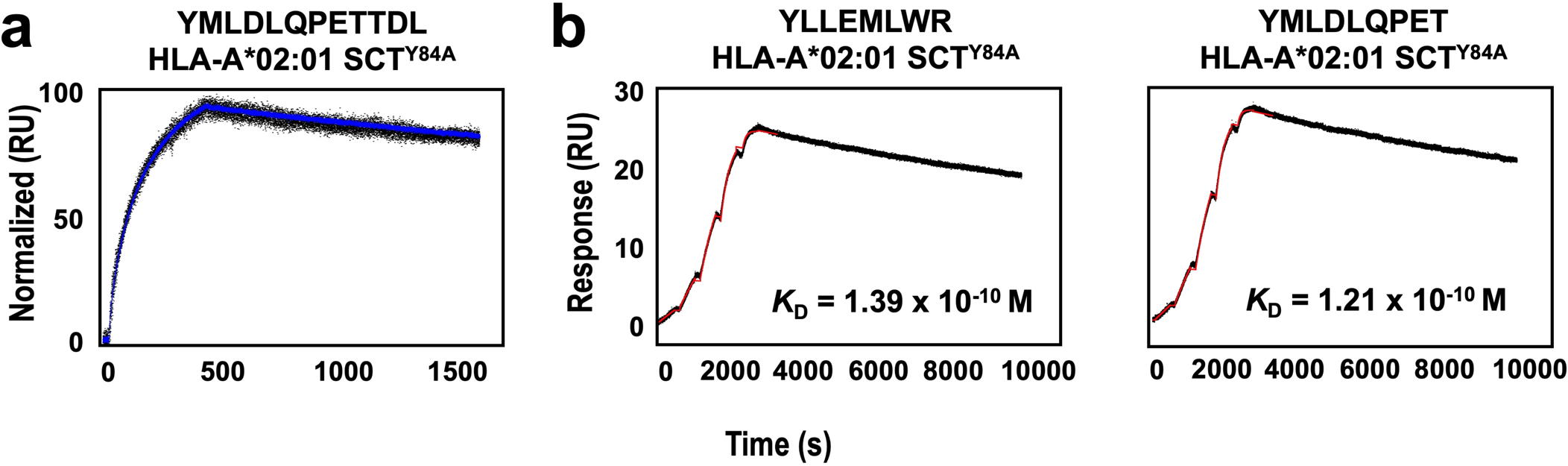
**

*Notes*:

SPR sensorgrams are shown for a) HLA-A*02:01 SCT^Y84A^ binding to W6/32 (in normalized RU); in-house recombinant W6/32 (blue), BioLegend W6/32 (black). b) Anti-SCT VHH, VHH-AD01, binding to two different, captured biotinylated SCTs (black), model fit (red). The two complexes differ in the incorporated peptide, as shown.
