# Supplementary material for: Effects of HLA single chain trimer design on peptide presentation and stability
Source: Front Immunol. 2023 May 3;14:1170462. doi: 10.3389/fimmu.2023.1170462 (PMC10189100; doi:10.3389/fimmu.2023.1170462)
Supplement: Supplementary file 2 [file DataSheet_2.docx]

**Supplementary Figure 2: Difference Fourier OMIT maps of SCT peptides.**

**
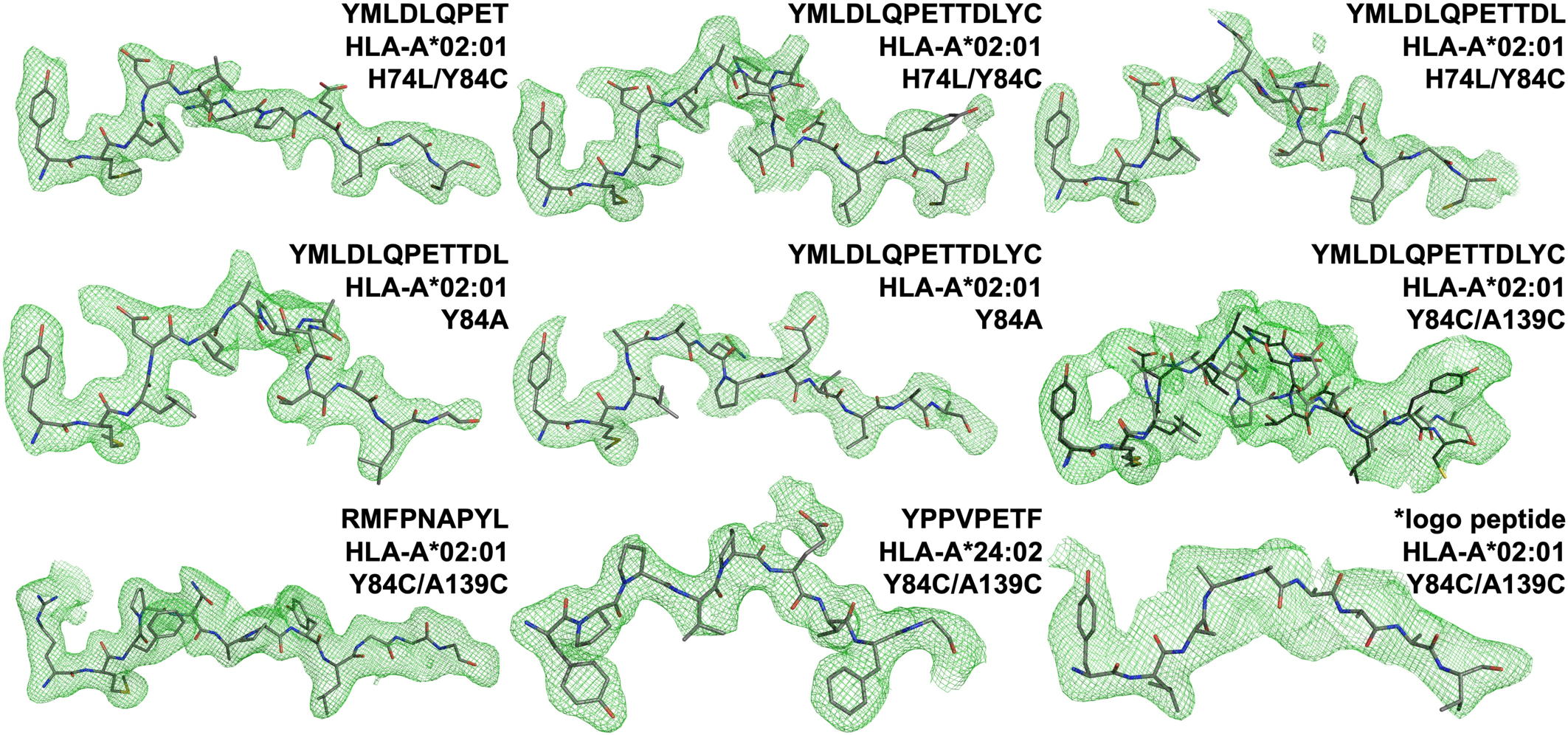
**

*Note*: electron density maps are shown in green mesh and bound peptides are shown in stick representation, colored by atom type. A “logo” peptide, a 9-mer peptide with A*02:01 consensus sequence anchors (YLAAAAAAV), was used to model the averaged set of bound endogenous peptides in the HLA*02:01^Y84C/A139C^ structure.
