# Supplementary material for: Effects of HLA single chain trimer design on peptide presentation and stability
Source: Front Immunol. 2023 May 3;14:1170462. doi: 10.3389/fimmu.2023.1170462 (PMC10189100; doi:10.3389/fimmu.2023.1170462)
Supplement: Supplementary file 3 [file DataSheet_3.docx]

**Supplementary Figure 3:** **Comparative reduced/ non-reduced SDS PAGE analyses of YML 14mer HLA-A*02:01 pHLA-I^RF^ and YML HLA-A*02:01 SCT constructs, including the glycosylated AML derived peptide, VLQELNVTV.**


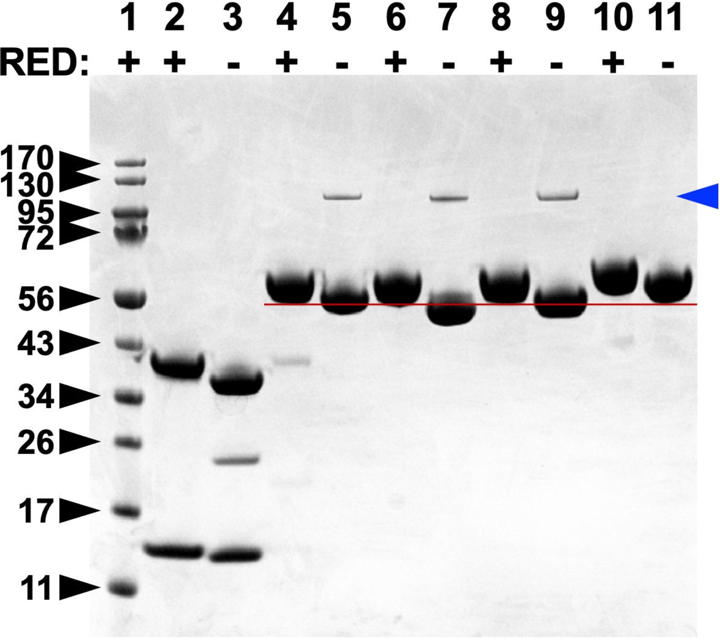


*Notes*: Lane 1: standards; lanes 2 and 3: YML 14-mer HLA-A*02:01 pHLA-I^RF^; lanes 4, 5: YML 14-mer SCT^Y84A^; lanes 6, 7: YML 14-mer SCT^H74L/Y84C^; lanes 8, 9: YML 14-mer SCT^Y84C/A139C^; lanes 10, 11: VLQELNVTV SCT^Y84A^**.** Addition of reducing agent is indicated by *plus signs* at the top of the gel. Bands at ~110 kDa (*blue arrow*) correspond to non-native dimers formed by oxidation of the cysteine-containing YML 14-mer peptide. SCTs containing peptides with N-linked glycosylation sites were shown to have a commensurate increase in molecular weight (*red line*) indicating that the presented peptides can be glycosylated during expression.
