# Supplementary material for: Effects of HLA single chain trimer design on peptide presentation and stability
Source: Front Immunol. 2023 May 3;14:1170462. doi: 10.3389/fimmu.2023.1170462 (PMC10189100; doi:10.3389/fimmu.2023.1170462)
Supplement: Supplementary file 4 [file DataSheet_4.docx]

**Supplementary datasheet: expression construct sequences, annotated:**

Generic SCT (Y84A) expression construct:

METDTLLLWVLLLWVPGSTG|***peptide***|GGGGSGGGGSGGGGSIQRTPKIQVYSRHPAENGKSNFLNCYVSGFHPSDIEVDLLKNGERIEKVEHSDLSFSKDWSFYLLYYTEFTPTEKDEYACRVNHVTLSQPKIVKWDRDMGGGGSGGGGSGGGGSGGGGS|GSHSMRYFFTSVSRPGRGEPRFIAVGYVDDTQFVRFDSDAASQRMEPRAPWIEQEGPEYWDGETRKVKAHSQTHRVDLGTLRG**A**YNQSEAGSHTVQRMYGCDVGSDWRFLRGYHQYAYDGKDYIALKEDLRSWTAADMAAQTTKHKWEAAHVAEQLRAYLEGTCVEWLRRYLENGKETLQRTDAPKTHMTHHAVSDHEATLRCWALSFYPAEITLTWQRDGEDQTQDTELVETRPAGDGTFQKWAAVVVPSGQEQRYTCHVQHEGLPKPLTLRWEHHHHHH

murine Igκ leader linkers β_2_m α-chain His_6_ **mutations**

Generic SCT (H74L Y84C) expression construct:

METDTLLLWVLLLWVPGSTG|***peptide***|GCGGSGGGGSGGGGSIQRTPKIQVYSRHPAENGKSNFLNCYVSGFHPSDIEVDLLKNGERIEKVEHSDLSFSKDWSFYLLYYTEFTPTEKDEYACRVNHVTLSQPKIVKWDRDMGGGGSGGGGSGGGGSGGGGS|GSHSMRYFFTSVSRPGRGEPRFIAVGYVDDTQFVRFDSDAASQRMEPRAPWIEQEGPEYWDGETRKVKAHSQT**L**RVDLGTLRG**C**YNQSEAGSHTVQRMYGCDVGSDWRFLRGYHQYAYDGKDYIALKEDLRSWTAADMAAQTTKHKWEAAHVAEQLRAYLEGTCVEWLRRYLENGKETLQRTDAPKTHMTHHAVSDHEATLRCWALSFYPAEITLTWQRDGEDQTQDTELVETRPAGDGTFQKWAAVVVPSGQEQRYTCHVQHEGLPKPLTLRWEHHHHHH

murine Igκ leader linkers β_2_m α-chain His_6_  **mutations**

Generic SCT (Y84C A139C) expression construct:

METDTLLLWVLLLWVPGSTG|***peptide***|GCGGSGGGGSGGGGSIQRTPKIQVYSRHPAENGKSNFLNCYVSGFHPSDIEVDLLKNGERIEKVEHSDLSFSKDWSFYLLYYTEFTPTEKDEYACRVNHVTLSQPKIVKWDRDMGGGGSGGGGSGGGGSGGGGS|GSHSMRYFFTSVSRPGRGEPRFIAVGYVDDTQFVRFDSDAASQRMEPRAPWIEQEGPEYWDGETRKVKAHSQTHRVDLGTLRG**C**YNQSEAGSHTVQRMYGCDVGSDWRFLRGYHQYAYDGKDYIALKEDLRSWTAADM**C**AQTTKHKWEAAHVAEQLRAYLEGTCVEWLRRYLENGKETLQRTDAPKTHMTHHAVSDHEATLRCWALSFYPAEITLTWQRDGEDQTQDTELVETRPAGDGTFQKWAAVVVPSGQEQRYTCHVQHEGLPKPLTLRWEHHHHHH

murine Igκ leader linkers β_2_m α-chain His_6_  **mutations**

Generic chimeric SCT expression construct:

METDTLLLWVLLLWVPGSTG|***peptide***|GGGGSGGGGSGGGGSIQ**R**TPQIQVYSRHPPENGKPNILNCYVTQFHP**SD**IEIQMLKNGKKIPKVEMSD**L**SFSKDWSFYILAHTEFTPTETDTYACRVKHASMAEPKTVYWDRDMGGGGSGGGGSGGGGSGGGGSGSHSMRYFFTSVSRPGRGEPRFIAVGYVDDTQFVRFDSDAASQRMEPRAPWIEQEGPEYWDGETRKVKAHSQTHRVDLGTLRG**A**YNQSEAGSHTVQRMYGCDVGSDWRFLRGYHQYAYDGKDYIALKEDLRSWTAADMAAQTTKHKWEAAHVAEQLRAYLEGTCVEWLRRYLENGKETLQRTDSPKAHVTYHPRSQVDVTLRCWALGFYPADITLTWQLNGEDLTQDMELVETRPAGDGTFQKWAAVVVPLGKEQNYTCHVHH**E**GLPEPLTLRWENLYFQ|GHHHHHHGLNDIFEAQKIEWHE

murine Igκ leader linkers murine β_2_m A2 α1α2 K^d^ α3 scission|site His_6_ Avi-Tag  **mutations**

Antibody W6/32 variable domain sequences:

V_H_: QVQLKQSGPGLVQPSQSLSLTCTVSGFSLTSYGVHWVRQPPGKGLEWLGVIWSGGSTDYNAAFISRLSIRKDNSKSQVFFKMNSLQADDTAIYYCARTFTTSTSAWFAYWGQGTLVTVS

V_L_:

SIVMTQTPKFLLVSAGDRVTITCKASQSVSNDVAWYQQKPGQSPKLLIYYASNRYTGVPDRFTGSGYGTDFTFTISTVQAEDLAVYFCQQDYSSPPWTFGGGTKLEIR

AD01 VHH expression construct:

METDTLLLWVLLLWVPGSTGDYKDEHHHHHHGGSQDSTSDLIPAPPLSKVPLQQNFQDNQFQGKWYVVGLAGNAILREDKDPQKMYATIYELKEDKSYNVTSVLFRKKKCDYWIRTFVPGSQPGEFTLGNIKSYPGLTSYLVRVVSTNYNQHAMVFFKKVSQNREYFKITLYGRTKELTSELKENFIRFSKSLGLPENHIVFPVPIDQCIDGGGSENLYFQ|GSEVKLVESGGGLVQPGGSLRLSCAASGSIFSINTMGWYRQTPGKQRDLVADISSGGSTKYGDSVKGRFTISRDNTKNTVYLQMNSLKPEDTAVYYCYGLSYSNDDYWGQGTQVTVSS

murine Igκ leader FLAG tag His_6_ linkers Scn fusion partner scission|site VHH CDR 1, 2, 3
