# Supplementary material for: Effects of HLA single chain trimer design on peptide presentation and stability
Source: Front Immunol. 2023 May 3;14:1170462. doi: 10.3389/fimmu.2023.1170462 (PMC10189100; doi:10.3389/fimmu.2023.1170462)
Supplement: Supplementary file 5 [file Table_1.docx]

**Supplementary Table 1: HLA allele/peptide complexes studied in this work.** Information incudes allele, type of construct (RF: *in vitro* refolded; SCT: single-chain trimer), any introduced mutations, the sequence of the incorporated peptide, NetMHCpan binding prediction, expression yield, and solution thermostability (Tm).

| **Allele** | **Construct** | **Mutation/s** | **Peptide** | **Predicted**  **Binding^†^** | **Yield^‡^** | **Tm (°C)** |
| --- | --- | --- | --- | --- | --- | --- |
| A*01:01 | RF | - | STDTLSSSTY | **+++** | RF | 54.1 ± 0.1 |
| A*01:01 | RF | - | YSGQGNNSY | **+++** | RF | 42.4 ± 0.0 |
| A*01:01 | SCT | Y84A | YVDFREYEYY | **+++** | *** | 42.0 ± 0.0 |
| A*02:01 | RF | - | KLLEIAPNC | **+** | RF | 59.8 ± 0.0 |
| A*02:01 | RF | - | SLLFLLFSL | **+++** | RF | 53.7 ± 0.4 |
| A*02:01 | RF | - | VLPVTVAEV | **+** | RF | 56.5 ± 0.4 |
| A*02:01 | SCT | Y84A | AIQDLCLAV | **+** | ~ | ~ |
| A*02:01 | SCT | H74L  Y84C | AIQDLCLAV | **+** | *** | 52.9 ± 0.4 |
| A*02:01 | SCT | Y84C  A139C | AIQDLCLAV | **+** | ND | 50.7 ± 0.1 |
| A*02:01 | SCT | Y84A | AIQDLCMAV | **+** | ~ | ~ |
| A*02:01 | SCT | Y84C | AIQDLCMAV | **+** | *** | 53.9 ± 0.1 |
| A*02:01 | SCT | H74L  Y84C | AIQDLCMAV | **+** | *** | 50.6 ± 0.4 |
| A*02:01 | SCT | Y84C  A139C | AIQDLCMAV | **+** | ND | 51.4 ± 0.2 |
| A*02:01 | SCT | Y84A | AIQDLCVAV | **+++** | ~ | ~ |
| A*02:01 | SCT | H74L  Y84C | AIQDLCVAV | **+++** | *** | 45.4 ± 0.3 |
| A*02:01 | SCT | Y84A | ALYVDSLFFL | **+++** | ~ | ~ |
| A*02:01 | SCT | - | ELAGIGILTV | **+** | **** | 44.1 ± 0.1 |
| A*02:01 | SCT | Y84A | ELAGIGILTV | **+** | **** | 45.5 ± 0.2 |
| A*02:01 | SCT | Y84C | ELAGIGILTV | **+** | **** | 53.3 ± 0.3 |
| A*02:01 | SCT | H74L | ELAGIGILTV | **+** | *** | 42.9 ± 0.5 |
| A*02:01 | SCT | H74L  Y84A | ELAGIGILTV | **+** | **** | 43.9 ± 0.1 |
| A*02:01 | SCT | H74L  Y84C | ELAGIGILTV | **+** | **** | 52.2 ± 0.4 |
| A*02:01 | SCT | Y84C  A139C | ELAGIGILTV | **+** | **** | 53.3 ± 0.3 |
| A*02:01 | SCT | Y84A | FLKANLPLL | **+++** | *** | ND |
| A*02:01 | SCT | Y84C | KLSAMQAHL | **+++** | ** | 52.0 ± 0.3 |
| A*02:01 | SCT | H74L  Y84C | KLSAMQAHL | **+++** | *** | 54.1 ± 0.1 |
| A*02:01 | SCT | Y84C  A139C | KLSAMQAHL | **+++** | ND | 51.1 ± 0.1 |
| A*02:01 | SCT | Y84A | LLMGTLGIV | **+** | *** | 44.1 ± 0.2  47.18^CD^ |
| A*02:01 | SCT | Y84C | LLMGTLGIV | **+** | ND | 54.3 ± 0.1 |
| A*02:01 | SCT | H74L  Y84C | LLMGTLGIV | **+** | ND | 54.0 ± 0.0 |
| A*02:01 | SCT | Y84C  A139C | LLMGTLGIV | **+** | ND | 52.5 ± 0.1 |
| A*02:01 | SCT | Y84C | LLNCIMDMV | **+** | ** | 51.5 ± 0.3 |
| A*02:01 | SCT | H74L  Y84C | LLNCIMDMV | **+** | *** | 55.0 ± 0.0 |
| A*02:01 | SCT | Y84C  A139C | LLNCIMDMV | **+** | *** | 50.1 ± 0.3 |
| A*02:01 | SCT | - | LQLPTLPLV | **+++** | ** | 43.9 ± 0.1 |
| A*02:01 | SCT | Y84A | LQLPTLPLV | **+++** | *** | 44.5 ± 0.2 |
| A*02:01 | SCT | Y84C | LQLPTLPLV | **+++** | **** | 53.5 ± 0.1 |
| A*02:01 | SCT | H74L | LQLPTLPLV | **+++** | **** | 50.5 ± 0.3 |
| A*02:01 | SCT | H74L  Y84A | LQLPTLPLV | **+++** | **** | 50.9 ± 0.1 |
| A*02:01 | SCT | H74L  Y84C | LQLPTLPLV | **+++** | **** | 56.9 ± 0.1 |
| A*02:01 | SCT | Y84C  A139C | LQLPTLPLV | **+++** | ND | 52.9 ± 0.2 |
| A*02:01 | RF | - | RMFPNAPYL | **+++** | RF | 55.0 ± 0.4 |
| A*02:01 | SCT | Y84A | RMFPNAPYL | **+++** | ~ | ~ |
| A*02:01 | SCT | H74L | RMFPNAPYL | **+++** | *** | 45.3 ± 0.2 |
| A*02:01 | SCT | H74L  Y84A | RMFPNAPYL | **+++** | *** | 45.3 ± 0.1 |
| A*02:01 | SCT | Y84C | RMFPNAPYL | **+++** | **** | 51.9 ± 0.1 |
| A*02:01 | SCT | H74L  Y84C | RMFPNAPYL | **+++** | **** | 53.9 ± 0.2 |
| A*02:01 | SCT | Y84C  A139C | RMFPNAPYL | **+++** | **** | 53.2 ± 0.1 |
| A*02:01 | SCT | Y84A | RVWFCMFLL | **×** | ~ | ~ |
| A*02:01 | SCT | - | SLLMWITQV | **+++** | ** | 44.7 ± 0.4 |
| A*02:01 | SCT | Y84A | SLLMWITQV | **+++** | ** | 45.6 ± 0.2 |
| A*02:01 | SCT | Y84C | SLLMWITQV | **+++** | *** | 55.0 ± 0.2 |
| A*02:01 | SCT | H74L | SLLMWITQV | **+++** | *** | 45.3 ± 0.3 |
| A*02:01 | SCT | H74L  Y84A | SLLMWITQV | **+++** | **** | 45.8 ± 0.0 |
| A*02:01 | SCT | H74L  Y84C | SLLMWITQV | **+++** | **** | 56.3 ± 0.1 |
| A*02:01 | SCT | Y84C  A139C | SLLMWITQV | **+++** | ND | 54.4 ± 0.4 |
| A*02:01 | SCT | - | SLLQHLIGL | **+++** | **** | 47.5 ± 0.6 |
| A*02:01 | SCT | Y84A | SLLQHLIGL | **+++** | **** | 45.5 ± 0.3 |
| A*02:01 | SCT | Y84C | SLLQHLIGL | **+++** | **** | 52.5 ± 0.1 |
| A*02:01 | SCT | H74L | SLLQHLIGL | **+++** | **** | 49.7 ± 0.4 |
| A*02:01 | SCT | H74L  Y84A | SLLQHLIGL | **+++** | **** | 47.7 ± 0.3 |
| A*02:01 | SCT | H74L  Y84C | SLLQHLIGL | **+++** | **** | 53.6 ± 0.3 |
| A*02:01 | SCT | Y84C  A139C | SLLQHLIGL | **+++** | ND | 55.1 ± 0.1 |
| A*02:01 | SCT | - | SVAPALALFPA | **×** | *** | 45.2 ± 0.2 |
| A*02:01 | SCT | Y84A | SVAPALALFPA | **×** | *** | 46.9 ± 0.1 |
| A*02:01 | SCT | Y84C | SVAPALALFPA | **×** | **** | 53.8 ± 0.0 |
| A*02:01 | SCT | H74L | SVAPALALFPA | **×** | *** | 43.6 ± 0.2 |
| A*02:01 | SCT | H74L  Y84A | SVAPALALFPA | **×** | *** | 43.8 ± 0.2 |
| A*02:01 | SCT | H74L  Y84C | SVAPALALFPA | **×** | **** | 52.0 ± 0.2 |
| A*02:01 | SCT | Y84C  A139C | SVAPALALFPA | **×** | ND | 54.2 ± 0.2 |
| A*02:01 | SCT | Y84A | TLGIVCPI | **×** | *** | 45.8 ± 0.0  50.46^CD^ |
| A*02:01 | SCT | Y84C  A139C | TLGIVCPI | **×** | ND | 46.9 ± 0.8 |
| A*02:01 | RF | - | VLDFAPPGA | **×** | RF | 54.9 ± 0.4 |
| A*02:01 | SCT | Y84A | VLHDDLLEA | **+++** | ** | 44.7 ± 0.3 |
| A*02:01 | SCT | Y84C | VLHDDLLEA | **+++** | *** | 54.7 ± 0.1 |
| A*02:01 | SCT | H74L  Y84C | VLHDDLLEA | **+++** | *** | 54.1 ± 0.1 |
| A*02:01 | SCT | Y84C  A139C | VLHDDLLEA | **+++** | ND | 54.0 ± 0.3 |
| A*02:01 | SCT | - | VLQELNVTV | **+++** | ** | 49.9 ± 0.8 |
| A*02:01 | SCT | Y84A | VLQELNVTV | **+++** | *** | 50.5 ± 0.2 |
| A*02:01 | SCT | Y84C | VLQELNVTV | **+++** | *** | 58.2 ± 0.2 |
| A*02:01 | SCT | H74L | VLQELNVTV | **+++** | *** | 52.6 ± 0.2 |
| A*02:01 | SCT | H74L  Y84A | VLQELNVTV | **+++** | *** | 52.8 ± 0.2 |
| A*02:01 | SCT | H74L  Y84C | VLQELNVTV | **+++** | *** | 59.5 ± 0.2 |
| A*02:01 | SCT | Y84C  A139C | VLQELNVTV | **+++** | ND | 56.0 ± 0.6 |
| A*02:01 | SCT | Y84A | YLLEMLWRL | **+++** | * | 50.9 ± 0.1 |
| A*02:01 | SCT | - | YMLDLQPE | **×** | *** | 43.3 ± 0.5 |
| A*02:01 | SCT | Y84A | YMLDLQPE | **×** | *** | 42.7 ± 0.5 |
| A*02:01 | SCT | Y84C | YMLDLQPE | **×** | *** | 50.6 ± 0.4 |
| A*02:01 | SCT | H74L | YMLDLQPE | **×** | **** | 46.3 ± 0.3 |
| A*02:01 | SCT | H74L  Y84A | YMLDLQPE | **×** | **** | 46.1 ± 0.3 |
| A*02:01 | SCT | H74L  Y84C | YMLDLQPE | **×** | **** | 52.3 ± 0.3 |
| A*02:01 | RF | - | YMLDLQPET | **+++** | RF | 58.5 ± 0.1 |
| A*02:01 | SCT | Y84A | YMLDLQPET | **+++** | *** | 45.1 ± 0.3  48.28^CD^ |
| A*02:01  K^d^ | χSCT | Y84A | YMLDLQPET | **+++** | *** | 46.1 ± 0.3 |
| A*02:01 | SCT | Y84C | YMLDLQPET | **+++** | **** | 53.2 ± 0.4 |
| A*02:01 | SCT | H74L | YMLDLQPET | **+++** | **** | 48.5 ± 0.2 |
| A*02:01 | SCT | H74L  Y84A | YMLDLQPET | **+++** | **** | 49.6 ± 0.5 |
| A*02:01 | SCT | H74L  Y84C | YMLDLQPET | **+++** | *** | 56.3 ± 0.1 |
| A*02:01 | SCT | - | YMLDLQPETT | **×** | **** | 47.3 ± 0.3 |
| A*02:01 | SCT | Y84A | YMLDLQPETT | **×** | **** | 47.7 ± 0.9 |
| A*02:01 | SCT | Y84C | YMLDLQPETT | **×** | **** | 55.2 ± 0.5 |
| A*02:01 | SCT | H74L | YMLDLQPETT | **×** | **** | 47.6 ± 0.4 |
| A*02:01 | SCT | H74L  Y84A | YMLDLQPETT | **×** | **** | 48.4 ± 0.4 |
| A*02:01 | SCT | H74L  Y84C | YMLDLQPETT | **×** | **** | 55.6 ± 0.2 |
| A*02:01 | SCT | - | YMLDLQPETTD | **×** | **** | 47.0 ± 0.4 |
| A*02:01 | SCT | Y84A | YMLDLQPETTD | **×** | **** | 47.4 ± 0.4 |
| A*02:01 | SCT | Y84C | YMLDLQPETTD | **×** | **** | 54.9 ± 0.2 |
| A*02:01 | SCT | H74L | YMLDLQPETTD | **×** | **** | 47.8 ± 0.4 |
| A*02:01 | SCT | H74L  Y84A | YMLDLQPETTD | **×** | **** | 48.3 ± 0.3 |
| A*02:01 | SCT | H74L  Y84C | YMLDLQPETTD | **×** | **** | 53.9 ± 0.1 |
| A*02:01 | RF | - | YMLDLQPETTDL | **+** | RF | 54.7 ± 0.1 |
| A*02:01 | SCT | Y84A | YMLDLQPETTDL | **+** | **** | 47.8 ± 0.0 |
| A*02:01 | SCT | Y84C | YMLDLQPETTDL | **+** | **** | 55.4 ± 0.0 |
| A*02:01 | SCT | H74L | YMLDLQPETTDL | **+** | **** | 49.9 ± 0.1 |
| A*02:01 | SCT | H74L  Y84A | YMLDLQPETTDL | **+** | **** | 49.6 ± 0.4 |
| A*02:01 | SCT | H74L  Y84C | YMLDLQPETTDL | **+** | **** | 57.4 ± 0.0 |
| A*02:01 | SCT | Y84C  A139C | YMLDLQPETTDL | **+** | ND | 55.9 ± 0.1 |
| A*02:01 | SCT | - | YMLDLQPETTDLY | **×** | **** | 48.1 ± 0.4 |
| A*02:01 | SCT | Y84A | YMLDLQPETTDLY | **×** | **** | 47.5 ± 0.3 |
| A*02:01 | SCT | Y84C | YMLDLQPETTDLY | **×** | **** | 53.5 ± 0.1 |
| A*02:01 | SCT | H74L | YMLDLQPETTDLY | **×** | **** | 50.1 ± 0.1 |
| A*02:01 | SCT | H74L  Y84A | YMLDLQPETTDLY | **×** | **** | 49.7 ± 0.4 |
| A*02:01 | SCT | H74L  Y84C | YMLDLQPETTDLY | **×** | **** | 54.7 ± 0.1 |
| A*02:01 | RF | - | YMLDLQPETTDLYC | **×** | RF | 62.2 ± 0.0 |
| A*02:01 | SCT | Y84A | YMLDLQPETTDLYC | **×** | *** | 48.1 ± 0.0 |
| A*02:01 | SCT | Y84C | YMLDLQPETTDLYC | **×** | *** | 55.3 ± 0.4 |
| A*02:01 | SCT | H74L | YMLDLQPETTDLYC | **×** | **** | 49.1 ± 0.6 |
| A*02:01 | SCT | H74L  Y84A | YMLDLQPETTDLYC | **×** | **** | 50.3 ± 0.4 |
| A*02:01 | SCT | H74L  Y84C | YMLDLQPETTDLYC | **×** | * | 53.5 ± 0.1 |
| A*02:01 | SCT | Y84C  A139C | YMLDLQPETTDLYC | **×** | *** | 56.2 ± 0.1 |
| A*02:01 | SCT | Y84C  A139C | YPPVPETF | **×** | * | ND^¶^ |
| A*11:01 | SCT | Y84A | AMFQDPQER | **+** | * | 54.8 ± 0.2 |
| A*11:01 | SCT | Y84A | GTTLEQQYNK | **+++** | *** | 51.0 ± 0.6 |
| A*11:01 | SCT | Y84A | SVYGTTLEQQY | **+++** | * | 51.0 ± 0.4 |
| A*11:01 | RF | - | SVYGTTLEKLTNK | **+++** | RF | 58.0 ± 0.0 |
| A*11:01 | RF | - | SVYGTTLEKLTN | **×** | RF | 50.9 ± 0.1 |
| A*11:01 | RF | - | SVYGTTLEKL | **+++** | RF | 57.4 ± 0.0 |
| A*11:01 | RF | - | SVYGTTLEK | **+++** | RF | 67.4 ± 0.4 |
| A*11:01 | SCT | Y84A | TTLEQQYNK | **+++** | * | 39.4 ± 0.0 |
| A*23:01 | RF | - | KFTPTTLPTF | **+++** | RF | 50.0 ± 0.0 |
| A*23:01 | RF | - | QFGYVSLFF | **+++** | RF | 51.7 ± 0.4 |
| A*23:01 | RF | - | RTYGYAFLFF | **+++** | RF | 53.0 ± 0.0 |
| A*24:02 | SCT | Y84A | AAATKYPLL | **×** | * | 53.1 ± 0.3 |
| A*24:02 | SCT | Y84A | DYLQYVLQI | **+++** | ** | 44.4 ± 0.0 |
| A*24:02 | SCT | Y84A | LFLIHTHARF | **+** | ** | 48.2 ± 0.0 |
| A*24:02  K^d^ | χSCT | Y84A | TYSAGIVQI | **+++** | * | 50.5 ± 0.2 |
| A*24:02 | SCT | Y84C  A139C | YPPVPETF | **+++** | *** | 56.3 ± 0.1 |
| B*07:02 | SCT | Y84A | IPRAHNRLV | **+++** | ~ | ~ |
| B*07:02 | SCT | Y84A | RPRTSCREA | **+++** | ** | ND |
| B*07:02 | SCT | Y84A | TPNQRQNVC | **+++** | ~ | ~ |
| B*40:01 | SCT | Y84A | QEAIQDLCL | **+** | ~ | ~ |
| B*40:01 | SCT | Y84A | REEMEVHEL | **+++** | * | 44.3 ± 0.1 |
| C*07:01 | RF | - | QFGYVSLFF | **×** | RF | 43.8 ± 0.0 |
| C*07:01 | RF | - | RTYGYAFLFF | **+** | RF | 50.6 ± 0.0 |
| C*07:01 | RF | - | RTYGYAFLFS | **×** | RF | 44.8 ± 0.0 |
| E*01:03 | SCT | Y84A | VMAPRTLFL | **+++** | *** | 45.9 ± 0.1 |
| G*01:02 | RF | - | KIPQVLVKL | **+++** | RF | 42.1 ± 0.0 |
| G*01:02 | SCT | Y84A | RIIPRHLQL | **+++** | * | 43.3 ± 0.1 |

Blocks of identical peptides are alternately colored blue or green.

*Notes*:

**^†^**: predicated binding was determined using the NetMHCpan 4.1 server ^1^ with default settings and is reported as: “**+++**”: strong binder, “**+**”: weak binder, and “**×**”: non-binder. Discordances between predicted binding and experimental expression are highlighted, with prediction false negatives in green and prediction false positives in orange. Predicted weakly-binding peptides that did not yield appreciable amounts of protein were not considered to be discordances.

**^‡^**: yields are reported as ~: too low for any experimental manipulations; *: <1 mg/L; **: 1 to 10 mg/L; ***: 11 to 40 mg/L; and ****: >40 mg/L.

ND: measurement was not done.

RF: Comparable, qualitative yields cannot be determined for *in vitro* refolded proteins.

CD: Tm Measurement done by circular dichroism.

Tms are reported as an average of three measurements ± the standard deviation.

^¶^: This T_m_ was not measured because the crystal structure showed a heterogenous mix of bound peptides.
