# Supplementary material for: Effects of HLA single chain trimer design on peptide presentation and stability
Source: Front Immunol. 2023 May 3;14:1170462. doi: 10.3389/fimmu.2023.1170462 (PMC10189100; doi:10.3389/fimmu.2023.1170462)
Supplement: Supplementary file 6 [file Table_2.docx]

**Supplementary Table 2: Crystallization conditions** (by vapor diffusion) for each deposited structure, by PDB accession code.

| **Accession code:** | **Well solution:** |
| --- | --- |
| 6APN | 0.1M MES, pH 6.5, 25% PEG 2000 MME |
| 6E1I | 0.1 M HEPES, pH 7.5, 25% PEG 6000 |
| 7SR0 | 200 mM HK_2_PO_4_, 20% PEG 3350 |
| 7SR3 | 200 mM K_2_SO_4_, 17% PEG 3350 |
| 7SR4 | 100 mM KCitrate, pH 6.5, 17.5% PEG 3350 |
| 7SSH | 200 mM NaCl, 100 mM HEPES, pH 7.0, 1.45 M (NH_4_)_2_SO_4_ |
| 7SR5 | 200 mM NH_4_Citrate, 14.5% PEG 3350 |
| 7SQP | 200 mM NH_4_Citrate, 20.0% PEG 3350 |
| 7STG | 100 mM Tris, pH 8.0, 200 mM Li_2_SO_4_, 1.1 M AmSO_4_ |
| 7ST3 | 100 mM Tris, pH 7.5, 200 mM Li_2_SO_4_, 1.6 M AmSO_4_ |
| 7SRK | 100 mM MES, pH 6.0, 200 mM KSCN, 18% Peg 3350 |
