# Supplementary material for: Effects of HLA single chain trimer design on peptide presentation and stability
Source: Front Immunol. 2023 May 3;14:1170462. doi: 10.3389/fimmu.2023.1170462 (PMC10189100; doi:10.3389/fimmu.2023.1170462)
Supplement: Supplementary file 7 [file Table_3.docx]

**Supplementary Table 3: Crystallographic data collection and refinement statistics**

| Accession code | 6E1I | 7SR0 | 7SQP | 7SR3 | 7SSH | 7SR4 | 7ST3 | 7SR5 | 6APN | 7STG | 7SRK |
| --- | --- | --- | --- | --- | --- | --- | --- | --- | --- | --- | --- |
| Allele | A*02:01  H-2K^d^ χSCT | A*02:01 | A*02:01 | A*02:01 | A*02:01 | A*02:01 | A*02:01 | A*02:01 | A*02:01 | A*02:01 | A*24:02 |
| Peptide | YML  9-mer | YML  9-mer | YML  12-mer | YML  12-mer | YML  14-mer | YML  14-mer | YML  14-mer | WT1 | LLMG-TLGIV | YPPV-PETF | YPPV-PETF |
| Mutations | Y84A | H74L  Y84C | Y84A | H74L  Y84C | Y84A | H74L  Y84C | Y84C  A139C | Y84C  A139C | Y84A | Y84C  A139C | Y84C  A139C |
| VHH | - | + | + | + | + | + | + | + | - | + | + |
| **Data collection** | | | | | | | | | | | |
| Space group | *P*22_1_2_1_ | *P*4_1_2_1_2 | *P*4_1_2_1_2 | *P*4_1_2_1_2 | *P*1 | *P*4_1_2_1_2 | *P*1 | *P*4_1_2_1_2 | *P*2_1_ | *P*1 | *P*2_1_2_1_2_1_ |
| Cell dimensions | | | | | | | | | | | |
| *a*, *b*, *c* (Å) | 52.47, 82.20, 107.2 | 117.7, 117.7, 261.8 | 118.0, 118.0, 262.7 | 118.1, 118.1, 262.2 | 117.7, 118.9, 275.0 | 117.9, 117.9, 262.3 | 117.8, 118.0, 273.5 | 117.5, 117.5, 261.3 | 50.36, 65.09, 130.3 | 117.59, 118.1, 273.7 | 69.92, 76.07, 227.0 |
| α, β, γ (°) | 90, 90,  90 | 90, 90, 90 | 90, 90, 90 | 90, 90, 90 | 77.60, 77.60, 90 | 90, 90, 90 | 102.5, 102.5 90 | 90, 90, 90 | 90, 96.25, 90 | 102.44, 102.4, 89.88 | 90, 90,  90 |
| Resolution (Å) | 50.0-  1.99  (2.02-1.99) | 50.0-  2.54 (2.59-2.54) | 50.0-  2.53  (2.59-2.53) | 50.0-  2.49  (2.54-2.49) | 50.0-  2.70  (2.75-2.70) | 50.0-  2.59  (2.64-2.59) | 50.0-  2.78  (2.85-2.78) | 50.0-  2.35  (2.39-2.35) | 50.00-2.22  (2.26-2.22) | 50.0-  2.70  (2.75-2.70) | 227-  2.50  (2.59-2.50) |
| *R*_merge_ (%) | 8.9 (44.3) | 10.7 (77.4) | 11.4 (53.7) | 11.5 (81.5) | 10.2 (58.5) | 12.9 (68.4) | 12.8 (53.3) | 7.5  (65.9) | 10.3 (37.0) | 5.5  (29.6) | 7.0  (37.1) |
| *I/*σ(*I*) | 33.9 (4.78) | 26.1 (2.23) | 33.9 (2.28) | 19.7 (2.25) | 15.9 (2.33) | 23.5 (2.79) | 9.76 (1.71) | 16.8 (1.67) | 12.5 (3.20) | 13.7 (1.71) | 29.5 (4.70) |
| *CC*_1/2_ | 0.981 (0.931) | 0.994 (0.856) | 0.997 (0.778) | 0.996 (0.847) | 0.986 (0.510) | 0.992 (0.848) | 0.998 (0.916) | 0.997 (0.772) | 0.931 (0.734) | 0.993 (0.739) | 0.999 (0.906) |
| Completeness (%) | 100 (100) | 97.6 (91.2) | 98.7 (90.5) | 99.9 (100) | 89.7 (89.0) | 100 (100) | 98.2 (97.5) | 99.4 (99.9) | 90.7 (74.0) | 94.7 (86.4) | 99.8 (100) |
| Redundancy | 7.2  (7.1) | 11  (9.4) | 9.3  (7.6) | 8.1  (8.2) | 2.3  (2.2) | 6.9  (6.9) | 1.8  (1.8) | 7.1  (6.5) | 3.1  (2.5) | 1.7  (1.7) | 9.6  (8.3) |
| **Refinement** | | | | | | | | | | | |
| Resolution (Å) | 44.9-2.00  (2.07-2.00) | 48.8-2.54  (2.63-2.54) | 49.0-2.53  (2.62-2.53) | 49.0-2.49  (2.58 -2.49) | 49.1-2.73  (2.83-2.73) | 48.9-2.59  (2.69-2.59) | 50.0-2.78  (2.88-2.78) | 48.7-2.35 (2.43-2.35) | 31.1-2.22  (2.30-2.22) | 48.9-2.70  (2.80-2.70) | 29.8-2.50  (2.59-2.50) |
| No. reflections | 32,268  (3,117) | 59,905  (5,425) | 61,220  (5,233) | 65,578  (6,332) | 33,1873  (28,511) | 58,080  (5,680) | 340,982  (29,320) | 76,473  (7,530) | 37,664  (3,208) | 362,304 (32,198) | 42,766  (4,213) |
| *R*_work_  *R*_free_ | 0.208  0.230 | 0.217  0.242 | 0.239  0.260 | 0.225  0.248 | 0.253  0.294 | 0.217  0.247 | 0.239  0.274 | 0.229  0.248 | 0.209  0.254 | 0.242  0.276 | 0.229  0.260 |
| No. atoms |  |  |  |  |  |  |  |  |  |  |  |
| Protein | 3,089 | 7,457 | 7,503 | 7,120 | 56,601 | 7,221 | 57,723 | 7,279 | 6,050 | 56,802 | 7,604 |
| Ion | - | 10 (PO_4_) | - | - | - | - | - | - | - | - | - |
| Water | 154 | 159 | 108 | 135 | 282 | 142 | 198 | 225 | 206 | 104 | 78 |
| *B* factors |  |  |  |  |  |  |  |  |  |  |  |
| Protein | 32.32 | 56.79 | 67.18 | 42.84 | 56.01 | 55.24 | 55.61 | 49.27 | 32.55 | 66.75 | 50.21 |
| Ion | - | 74.73 | - | - | - | - | - | - | - | - | - |
| Water | 37.04 | 48.01 | 56.35 | 34.71 | 42.08 | 46.90 | 40.90 | 42.78 | 29.60 | 45.73 | 35.28 |
| R.m.s. deviations | | | | | | | | | | | |
| Bond  lengths  (Å) | 0.007 | 0.002 | 0.002 | 0.002 | 0.004 | 0.005 | 0.004 | 0.002 | 0.009 | 0.003 | 0.005 |
| Bond  angles  (°) | 1.14 | 0.52 | 0.50 | 0.51 | 0.53 | 0.61 | 0.96 | 0.49 | 1.11 | 0.52 | 0.59 |

*Notes*:

Values in parentheses are for highest-resolution shell.

Peptides: YML 9-mer: YMLDLQPET; YML 12-mer: YMLDLQPETTDL; YML 14-mer: YMLDLQPETTDLYC; WT1: RMFPNAPYL
