# Supplementary material for: Effects of HLA single chain trimer design on peptide presentation and stability
Source: Front Immunol. 2023 May 3;14:1170462. doi: 10.3389/fimmu.2023.1170462 (PMC10189100; doi:10.3389/fimmu.2023.1170462)
Supplement: Supplementary file 8 [file Table_4.docx]

**Supplementary Table 4: β_2_m/VHH intermolecular contacts**.

| β2m residue [atom] | Distance  (Å) | VHH residue [atom] |
| --- | --- | --- |
| ASN  42 [N] | 3.78 | ASN 102 [OD1] |
| GLY  43 [N] | 2.70 | ASN 102 [OD1] |
| ARG  45 [NH1] | 3.05 | ASP  50 [OD1] |
| ARG  45 [NH1] | 3.27 | ASP  50 [OD2] |
| ARG  45 [NH2] | 3.01 | ASP  50 [OD2] |
| ARG  81 [NH1] | 3.08 | TYR  96 [OH] |
| ASN  83 [ND2] | 2.79 | ARG  45 [O] |
| LYS  94 [NZ] | 2.92 | ASP 103 [OD1] |
| LYS  94 [NZ] | 2.89 | SER 101 [O] |
| ASN  42 [O] | 2.87 | ASN  32 [ND2] |
| GLY  43 [O] | 3.33 | THR  33 [N] |
| GLY  43 [O] | 2.80 | THR  33 [OG1] |
| GLU  44 [OE1] | 3.43 | ASN  32 [ND2] |
| GLU  77 [O] | 3.23 | ASN 102 [ND2] |
| GLU  77 [OE2] | 2.78 | SER 101 [OG] |
| THR  86 [O] | 3.38 | LYS  43 [NZ] |
| SER  88 [O] | 3.05 | ARG  45 [NE] |
| ARG  45 [NH1] | 3.05 | ASP  50 [OD1] |
| ARG  45 [NH1] | 3.27 | ASP  50 [OD2] |
| ARG  45 [NH2] | 3.93 | ASP  50 [OD1] |
| ARG  45 [NH2] | 3.01 | ASP  50 [OD2] |
| LYS  94 [NZ] | 2.92 | ASP 103 [OD1] |
| LYS  94 [NZ] | 3.64 | ASP 103 [OD2] |
| GLU  36 [OE2] | 3.95 | LYS  58 [NZ] |
| ASP  38 [OD1] | 3.96 | LYS  58 [NZ] |

*Notes*:

PISA interface analysis (PDBePISA) between chain C (β_2_m) and D (VHH), PDB 7SQP; Hydrogen bonds shown in black, salt bridges shown in red.
